# Reconstruction of the raffinose family oligosaccharide pathway in maize improves heat stress tolerance

**DOI:** 10.1007/s44154-026-00326-0

**Published:** 2026-07-16

**Authors:** Zhongchun Dong, Tao Li, Xudong Li, Dan Li, Xianfei Shi, Jiahao Chai, Lynnette M. A. Dirk, A. Bruce Downie, Tianyong Zhao

**Affiliations:** 1 State Key Laboratory of Crop Stress Resistance and High-Efficiency Production, College of Life Sciences, Northwest A&F University, Yangling, Shaanxi 712100 China; 2 State Key Laboratory of Wheat and Maize Crop Science, Collaborative Innovation Center of Henan Grain Crops, College of Life Science, Henan Agricultural University, Zhengzhou, Henan 450002 China; 3 College of Agriculture and Bioengineering (Peony College), Heze University, 2269 Daxue Road, Heze, Shandong 274015 People’s Republic of China; 4 Department of Horticulture, Seed Biology, Martin-Gatton College of Agriculture, Food and Environment, University of Kentucky, Lexington, KY 40546 USA

**Keywords:** Raffinose family oligosaccharides (RFOs), Maize, Arabidopsis, Stachyose synthase, Stachyose, Heat tolerance

## Abstract

Raffinose family oligosaccharides (RFOs), primarily raffinose and stachyose, are crucial for plant abiotic stress tolerance. Raffinose is synthesized by RAFFINOSE SYNTHASE (RAFS) from galactinol and sucrose, whereas stachyose can be produced by STACHYOSE SYNTHASE (STS) from galactinol and raffinose. While Arabidopsis possesses functional *RAFS* and *STS* genes and produces both raffinose and stachyose, maize lacks both a functional *STS* gene and endogenous stachyose. Here, we show that Arabidopsis* STACHYOSE SYNTHASE* (*AtSTS*) is induced by heat stress, and its overexpression and subsequent stachyose accumulation enhance heat tolerance. To extend the RFOs pathway in maize, we introduced the *AtSTS* expression cassette into the maize genome. The resulting transgenic maize lines accumulated stachyose and exhibited improved heat tolerance. Under heat stress, stachyose amounts in these lines increased significantly, likely due to the induction of endogenous *ZmRAFS* and subsequent protein and raffinose production, the latter serving as the STS substrate. Compared with wild-type controls, transgenic maize seedlings showed reduced leaf damage, higher survival, and lower ion leakage under high-temperature conditions. The application of exogenous stachyose similarly improved heat tolerance in wild-type maize, providing further evidence of its protective function. Moreover, *AtSTS* overexpression reduced reactive oxygen species (ROS) accumulation, indicating that stachyose helps mitigate oxidative damage. These findings establish stachyose as a contributor to heat stress resilience and propose a promising strategy for improving crop tolerance to increasing global temperatures.

**Supplementary Information:**

The online version contains supplementary material available at 10.1007/s44154-026-00326-0.

## Introduction

Increases in global temperatures pose a significant and growing threat to agricultural productivity worldwide (Zhao et al. 2017). Heat stress disrupts essential physiological processes in plants, leading to reduced growth, impaired development, and ultimately, yield losses (Cheikh and Jones 1994). To mitigate these adverse effects, plants have evolved a sophisticated array of biochemical and molecular adaptations. Among these, the accumulation of specialized protective metabolites, such as the raffinose family oligosaccharides (RFOs), plays a vital role in conferring tolerance to various abiotic stresses, including heat, cold, and drought (Gu et al. 2016; Selvaraj et al. 2017). RFOs, which include raffinose, stachyose, and verbascose, are non-reducing sugars synthesized in the cytosol (Peterbauer and Richter 2001). They are believed to contribute to stress resilience through multiple mechanisms, acting as compatible solutes for osmotic adjustment, stabilizing cellular membranes (Cacela and Hincha 2006), and potentially serving as antioxidants to scavenge reactive oxygen species (ROS) (Nishizawa et al. 2008). The biosynthesis of RFOs proceeds in a stepwise manner. Raffinose, the foundational member, is synthesized from sucrose and galactinol by the enzyme RAFFINOSE SYNTHASE (RAFS; EC 2.4.1.82 (Peterbauer et al. 2002)). Stachyose, the next higher oligomer, can then be produced by STACHYOSE SYNTHASE (STS, EC 2.4.1.67), which adds another galactosyl unit from galactinol to raffinose (the galactinol dependent pathway) (Gangl et al. 2015), or, in some species, can be synthesized from two raffinose molecules by the enzyme GALACTAN:GALACTAN GALACTOSYLTRANSFERASE (the galactinol independent pathway) (Haab and Keller 2002).

Interestingly, the presence and composition of RFOs vary considerably among plant species (Kuo et al. 1988), suggesting evolutionary divergence in stress adaptation strategies. *Arabidopsis thaliana*, possesses an RFO-synthesis pathway that includes both functional *RAFS* and *STS* genes with a corresponding accumulation of raffinose, stachyose, and verbascose in seeds (Li et al. 2017). In contrast, many important cereal crops, including maize (*Zea mays*), present a different metabolic profile (Li et al. 2017). Maize accumulates raffinose but lacks detectable levels of stachyose (Wen et al. 2018), suggesting a discrepancy correlated with the absence of a functional *STS* gene in its genome. This raises critical questions about the specific functional contribution of stachyose to heat tolerance and whether the RFO pathway in maize represents a metabolic limitation for heat stress adaptation. From an applied perspective, metabolic engineering to reconstitute "missing" protective pathways offers a promising strategy for enhancing crop climate resilience. If stachyose is a determinant of heat tolerance, its engineered production in a non-accumulating species like maize could provide a direct route to improved performance under high-temperature conditions. Additionally, this might be accomplished without a yield penalty (Gu et al. 2016).

In this study, we hypothesized that stachyose is a mediator of heat tolerance and that introducing the capability for its synthesis into maize would enhance the plant's heat tolerance. We first confirmed in Arabidopsis that its endogenous *STS* gene (*AtSTS*) is heat-inducible and that its overexpression boosts heat tolerance. We then introduced the *AtSTS* gene into the maize genome to engineer a further step of oligomerization in the RFOs pathway. We rigorously evaluated the resulting transgenic maize lines, assessing stachyose accumulation, physiological and biochemical responses to heat stress, and overall plant and yield parameters. Furthermore, we tested the protective effect of exogenously applied stachyose on Zong31 (Z31) maize. Our findings collectively establish stachyose as a metabolite that could increase protection against heat stress and demonstrate the viability of pathway engineering to bolster crop tolerance in a warming world.

## Results

### Stachyose and STACHYOSE SYNTHASE are present in Arabidopsis but absent in maize

When present, stachyose is synthesized by the addition of a galactosyl group to raffinose, catalyzed either by STACHYOSE SYNTHASE (STS) via a galactinol-dependent pathway or by GALACTAN:GALACTAN GALACTOSYLTRANSFERASE (GGT; EC 2.4.1.22) via a galactinol-independent pathway (Haab and Keller 2002).

**Fig. 1:**
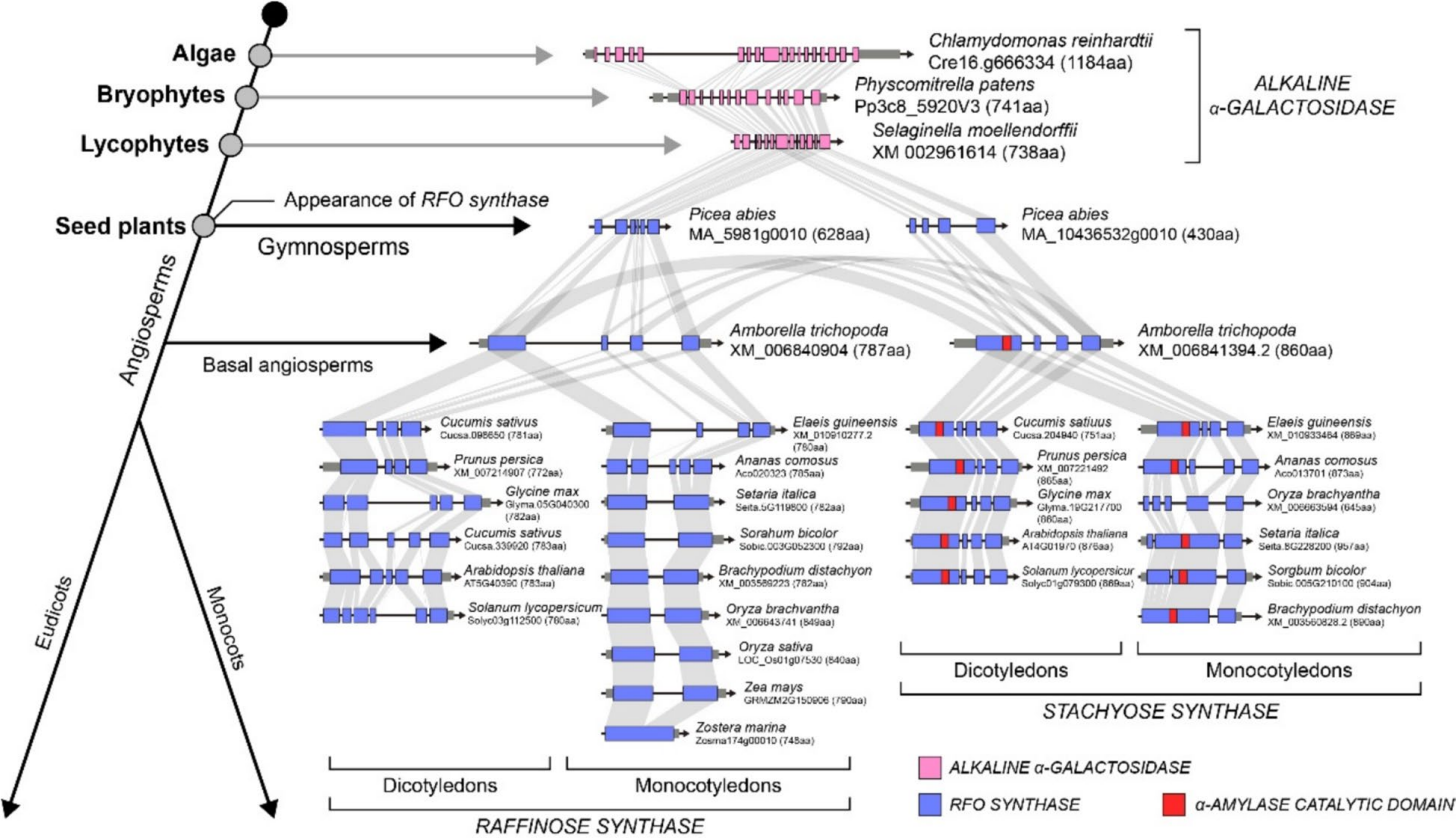
Evolutionary diversification and structural conservation of Raffinose family oligosaccharide (RFO) Synthase-related genes across plant lineages. A simplified phylogenetic framework of major plant groups is shown on the left, including Algae, Bryophytes, Lycophytes, Seed plants, Gymnosperms, Basal angiosperms, Eudicots, and Monocots. The emergence of Raffinose family oligosaccharide (RFO) Synthase genes during seed plant evolution is indicated. Gene structures of representative homologs for each lineage are displayed on the right. Pink and blue boxes represent coding regions of Alkaline α-Galactosidase (AGA) and RFO Raffinose Synthase, respectively, whereas gray boxes indicate untranslated regions (UTRs). Red boxes indicate the coding regions of Alpha-amylase catalytic domain identified in Stachyose Synthase (STS). The length of the encoded protein is indicated beside each gene model. Gray connecting ribbons represent homologous relationships among exons of genes across species

To investigate the evolutionary distribution of raffinose family oligosaccharide (RFO) biosynthetic enzymes, AtRAFS, AtSTS, and ZmRAFS were used as query sequences to search against 62 plant genomes. A total of 391 homologous protein sequences were identified, among which 135 sequences clustered with functionally characterized RFO biosynthetic enzymes in phylogenetic analyses. Notably, all of these sequences were derived from seed plants (Supplementary Fig. 1). Although homologous sequences were identified in chlorophytes, bryophytes, and pteridophytes, phylogenetic analysis revealed that these sequences were more closely related to ALKALINE α-GALACTOSIDASES (AGA) rather than bona fide RFO SYNTHASES (Supplementary Fig. 1). These findings suggest that RAFS and STS likely originated from an ancestral AGA through evolutionary divergence. In the gymnosperm representative species *Picea abies*, putative RAFS and STS homologs (MA_5981g0010 and MA_10436532g0010) were identified, which clustered into the RAFS and STS clades, respectively (Supplementary Fig. 1 and Fig. 1). In the basal angiosperm *Amborella trichopoda*, the STS homolog (XM_006841394.2) contains an additional N-terminal extension compared to its counterpart in *Picea abies*, including a predicted α-AMYLASE catalytic domain (Fig. 1). Furthermore, STS homologs were identified in most angiosperm lineage. However, notable exceptions were observed in grasses (Gramineae), including maize and *Oryza sativa* (rice), in which only RAFS homologs were detected (Supplementary Fig. 1 and Fig. 1). Consistently, no STS homologs were identified in the genomes of wild relatives of maize, including *Zea mays ssp. parviglumis*, *Zea mays ssp. mexicana*, and *Zea mays ssp. huehuetenagensis*, based on searches in the MaizeGDB database. In addition, RAFS protein sequences from these wild subspecies were identical to those of the B73 inbred line. In contrast, STS is retained in other domesticated grasses, such as *Setaria italica* and *Sorghum bicolor* (Supplemental Fig. 1 and Fig. 1). Together, these results suggest that STS originated prior to the divergence of gymnosperms and angiosperms, and was subsequently lost in the maize lineage. Importantly, the absence of STS in both cultivated maize and its wild relatives indicates that this gene loss likely occurred early in maize evolution, rather than during domestication.

**Fig. 2:**
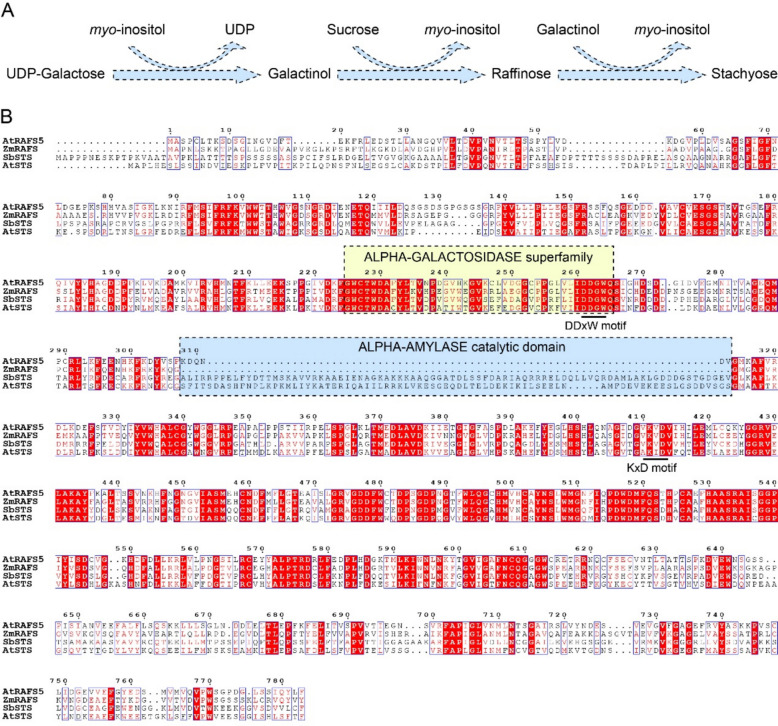
Schematic of stachyose biosynthesis and alignment of Stachyose Synthase (STS) and Raffinose Synthase (RAFS) amino acid sequences. **A** Schematic diagram of stachyose anabolism. **B** The full protein sequences alignment among AtSTS (NP_192106.3), SbSTS (XP_002451238.1), AtRAFS5 (NP_198855.1), ZmRAFS (NP_001354805.1). Conserved domains of the Alpha-Galactosidase superfamily, the Alpha-Amylase catalytic domain specific to STS proteins, as well as the previously reported DDxW and KxD motifs, are highlighted

Since maize lacks a functional STS (Fig. 2A) and does not accumulate stachyose (Li et al. 2017), the *Arabidopsis thaliana STS* gene (At4g01970, *AtSTS*) was selected for introduction into this non-accumulating species to investigate the role of stachyose under abiotic stress. The *AtSTS* coding region (2628 bp) encodes a protein of 876 amino acids (Gangl et al. 2015). An amino acid sequence homology alignment was performed comparing AtSTS, SbSTS, AtRAFS5 and ZmRAFS (Fig. 2B). The α-AMYLASE catalytic domain (AC domain), essential for stachyose synthesis (Li et al. 2017), was found only in AtSTS and SbSTS but not in RAFS (Fig. 2B).

### *AtSTS*-overexpression enables stachyose synthesis and accumulation in maize leaves under heat stress

Based on the Arabidopsis TAIR database, *AtSTS* expression is upregulated under heat stress. This response was confirmed by quantitative RT-PCR and Western blot analyses, which showed short-term heat-induced accumulation of both *AtSTS* mRNA and protein in Arabidopsis seedling leaves (Supplementary Fig. 2). To investigate the role of stachyose in Arabidopsis heat stress responses, the heat tolerance of three 3-wk-old *AtSTS*-overexpressing Arabidopsis lines (OE*AtSTS*#1, #5, #9) was assessed. Under normal growth conditions, no morphological differences were observed between the *AtSTS*-overexpressing seedlings and the Col-0 control (top panel, Supplementary Fig. 3 A). After 36 h of heat stress at 43 °C, leaves of Col-0 plants began to yellow, with severe damage and wilting, whereas leaves of some *AtSTS*-overexpressing plants remained green and showed less injury (bottom panel, Supplementary Fig. 3 A). Following a 6-d recovery, two thirds of the control plants had died, while more than 50% of the *AtSTS*-overexpressing plants resumed growth (bottom panel, Supplementary Fig. 3 A and B). Additionally, the ion leakage across the plasma membrane in leaves of the *AtSTS*-overexpressing plants was markedly lower compared with that in leaves of Col-0 plants (Supplementary Fig. 3 C).

**Fig. 3:**
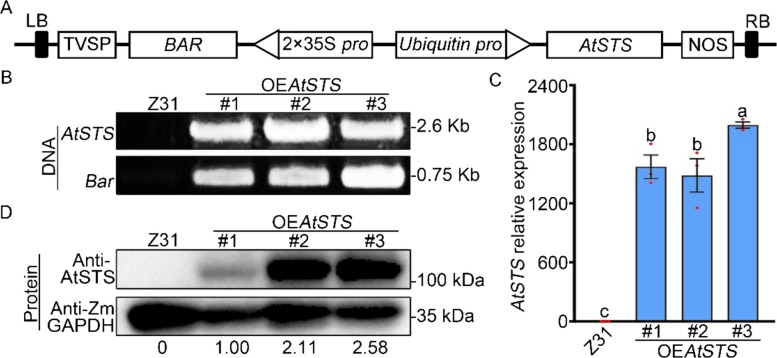
Identification of transgenic *AtSTS* maize lines. **A** Schematic representation of the construct used for maize transformation. 2 × 35S *pro*, 2 × CaMV 35S promoter; *BAR*, bialaphos resistance gene; TVSP, T7 terminator; *Ubiquitin pro*, *U*biquitin promoter; NOS, terminator; RB, right border; LB, left border. **B** Characterization of *AtSTS*-overexpressing (OE*AtSTS*) maize lines. PCR amplification of the transformed *AtSTS* and *BAR* genes in Z31 and OE*AtSTS* maize plants. **C** Quantitative RT-PCR characterization of the *AtSTS* mRNA accumulation in Z31 and OE*AtSTS* maize seedlings. *ZmGAPDH* accumulation was used as the internal control. Each red dot represents one biological replicate; there are three biological replicates for each line. Values are means ± SEM. Different letters indicate significant differences (ANOVA followed by Duncan's test, *P* < 0.05). **D** Western blot analysis of the AtSTS protein accumulation in Z31 and OE*AtSTS* maize seedlings. ZmGAPDH protein was used as the internal control. Band volumes (arbitrary units) were assessed using Image J. The AtSTS amounts were normalized by ZmGAPDH amounts, represented by the band volume ratios under the blots

To investigate the role of stachyose in maize under heat stress, an *AtSTS-*overexpression vector housing a gene whose protein product has been demonstrated to produce stachyose (Gangl et al 2015), was constructed and introduced into a maize inbred line, Z31. Expression of *AtSTS* was driven by the *Ubiquitin* promoter (*pUBI*) (Fig. 3A). The *AtSTS*-overexpressing plants were backcrossed for five generations with the recurrent parent Z31 in the field to eliminate the background variation induced by cell culture and transformation, followed by three generations of self-pollination to generate homozygous transgenic lines (Supplementary Fig. 4). This breeding strategy produced three stably inherited overexpression lines (OE*AtSTS*#1, #2, #3), in which transgene integration and expression were confirmed by PCR, RT-qPCR, and Western blot analyses (Fig. 3B-D).

**Fig. 4:**
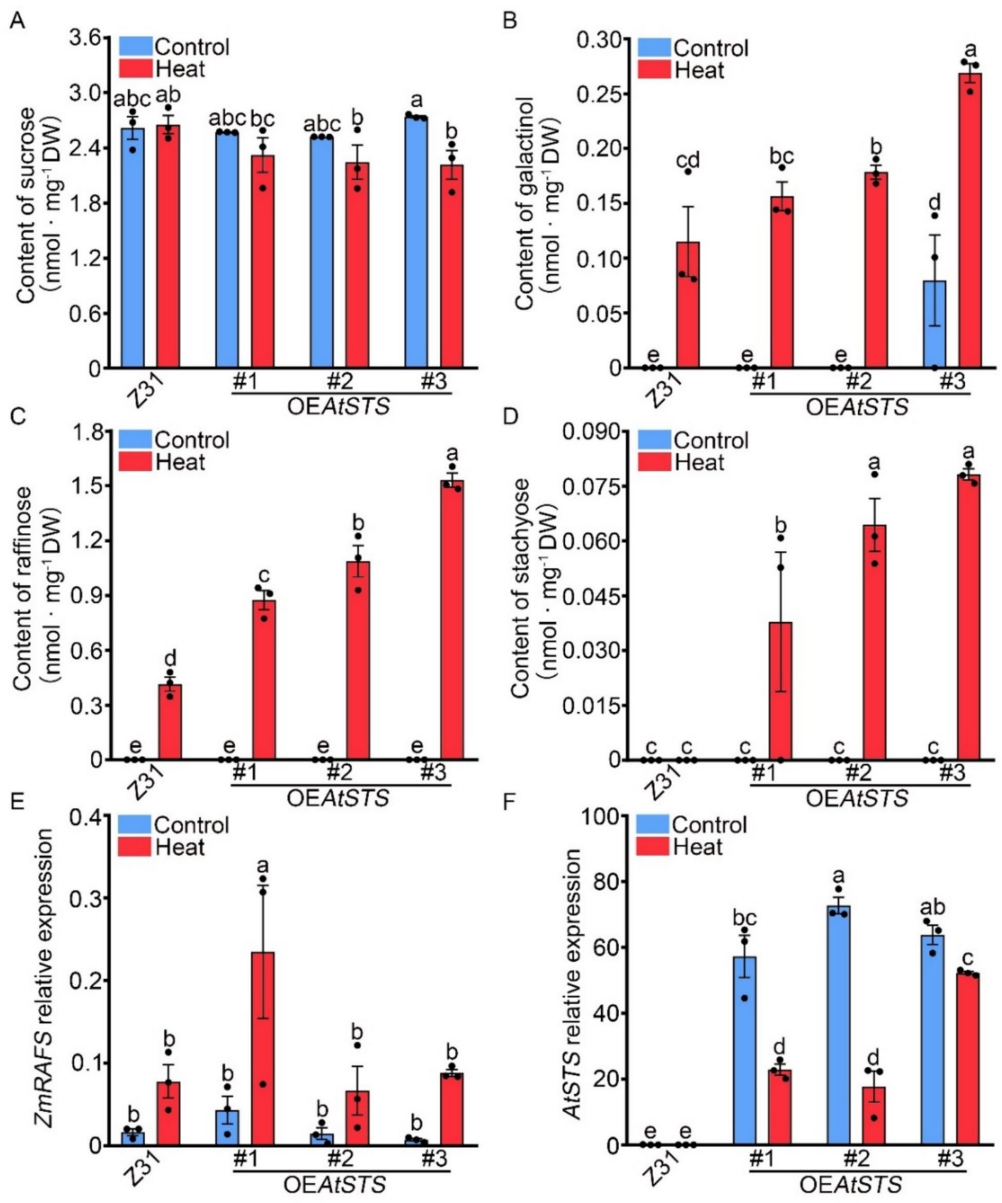
Changes in oligosaccharide components in leaves of the maize inbred line Z31 and *AtSTS*-overexpressing maize without and with heat stress treatment. **A**-**D** Comparison of leaf sugar content between Z31 and *AtSTS*-overexpressing (OE*AtSTS*) maize plants that were treated with or without heat stress. Mature leaves on plants growing at 28℃ were treated at 28℃ or 43℃ for 4 h, then were used for determination of (**A**) sucrose, (**B**) galactinol, (**C**) raffinose, and (**D**) stachyose content. Values are means ± SEM (*n* = 3). Different letters indicate significant differences (Duncan's test, *P* < 0.05). **E**, **F** Real time RT-PCR analysis of the *ZmRAFS* (**E**) and *AtSTS* (**F**) mRNA accumulation in leaves of maize variety Z31 and OE*AtSTS* maize without and with heat stress treatment. *ZmGAPDH* accumulation was used as the internal control. Values are means ± SEM (*n* = 3). Different letters indicate significant differences (Duncan's test, *P* < 0.05)

To investigate the role of stachyose in the maize response to high-temperature stress, we analyzed soluble sugar contents and the expression of related genes in the leaves of three-leaf-stage seedlings of the maize line Z31 and three independent *AtSTS*-overexpressing lines under normal conditions (28 °C) and after heat stress treatment (43 °C). Under normal growth conditions, no significant differences were observed in the contents of sucrose, raffinose, or stachyose among the lines, with the exception of galactinol content, which was significantly higher in the OE*AtSTS*#3 line than in the other lines (Fig. 4A-D). After 4 h of heat stress, the sucrose content increased in Z31 but decreased in all three overexpression lines (Fig. 4A). Concurrently, the contents of galactinol and raffinose significantly increased in all lines following heat stress (Fig. 4B-C). Stachyose was detected exclusively in the *AtSTS*-overexpressing lines, and its content increased significantly after heat stress, while remaining undetectable in Z31 throughout the experiment (Fig. 4D). This stress-induced stachyose accumulation correlated with a marked upregulation of the *ZmRAFS*, whose protein product likely expanded the raffinose pool available for stachyose synthesis via the galactinol-dependent pathway (Fig. 4E). Although the accumulation of *AtSTS* transcripts was slightly suppressed by heat, the corresponding protein likely remained sufficiently active to convert the heat-induced increase in raffinose into detectable amounts of stachyose (Fig. 4F). Raffinose levels also increased in Z31 upon heat stress; however, the absence of detectable stachyose accumulation in this line suggests that maize lacks a functional galactinol-independent pathway for stachyose synthesis. Together, these findings suggest that stachyose accumulation in *AtSTS*-overexpressing plants is limited by raffinose availability, and that heat stress relieves this limitation by inducing *ZmRAFS* expression, thereby redirecting carbon flux toward stachyose synthesis. The substantial accumulation of stachyose in heat-stressed, but not unstressed transgenic maize leaves implies a functional role for stachyose in heat tolerance.

### Overexpression of *AtSTS* enhances heat tolerance in maize

**Fig. 5:**
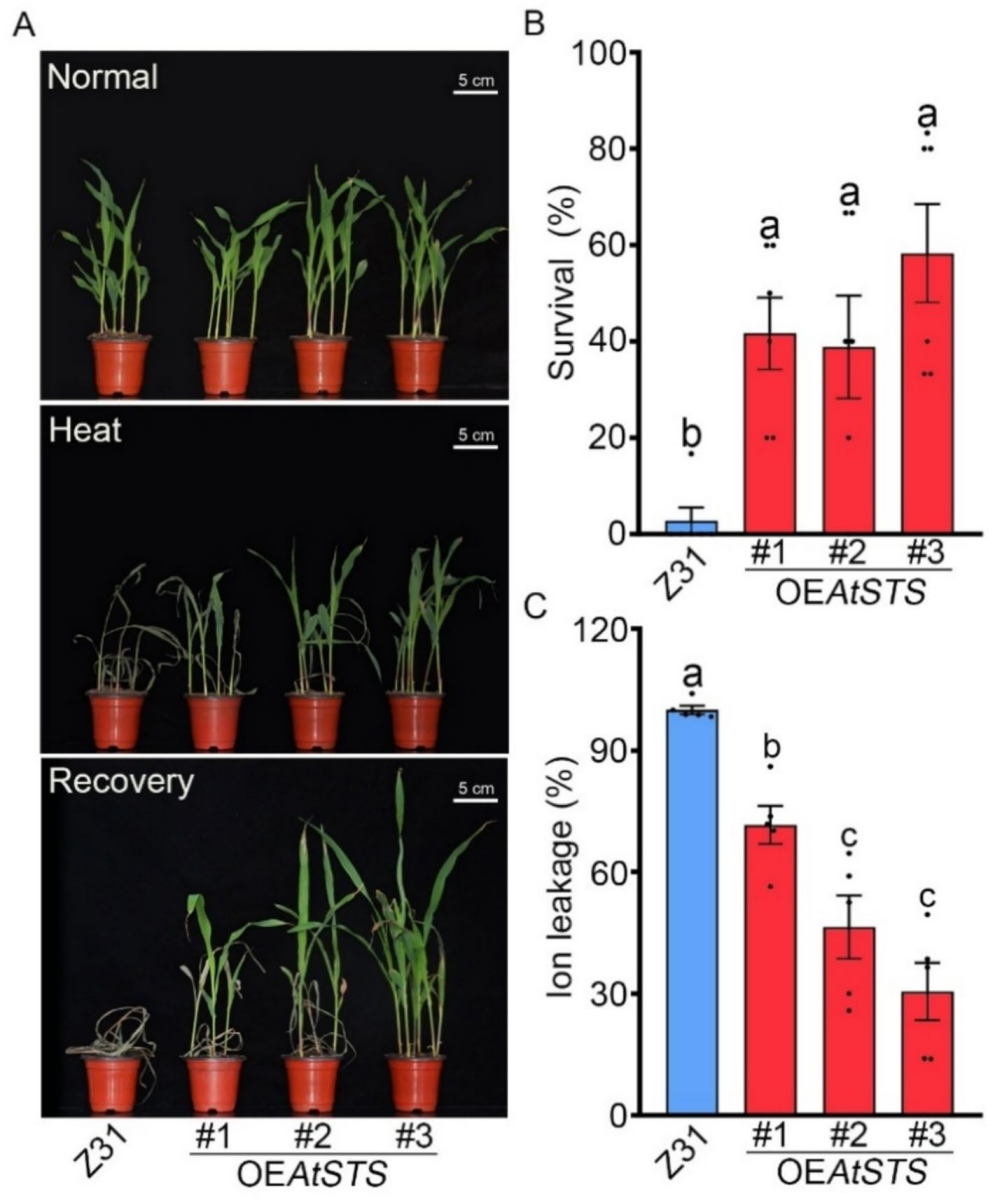
Overexpression of *AtSTS* enhances the maize seedlings heat tolerance. **A** Morphology comparisons between non-transgenic Z31 plants and *AtSTS*-overexpressing (OE*AtSTS*) plants before heat stress (top panel), after 45℃ heat stress of 24 h (middle panel) and 7 d after recovering (bottom panel). The scale bar = 5 cm. **B** Comparison of the survival between control non-transgenic Z31 plants and OE*AtSTS* plants after heat stress. Each black dot represents one biological replicate (of five plants each); there are six biological replicates for each line. *n* = 6 independent pots, each with 5 plants.Values are means ± SEM. Different letters indicate significant differences (Duncan's test, *P* < 0.05). **C** Comparison of the plasma membrane ion leakage between control non transgenic Z31 plants and OE*AtSTS* plants after heat stress. The second leaf from different plants was collected for testing of the electrolyte leakage. Each black dot represents one biological replicate (of five plants each); there are five biological replicates for each line. *n* = 5 independent pots, each with 5 plants. Values are means ± SEM. Different letters indicate significant differences (Duncan's test, *P* < 0.05)

Heat tolerance was also evaluated in V3-stage seedlings of the three *AtSTS*-overexpressing maize lines. Under normal conditions, no morphological differences were apparent between transgenic and non-transgenic Z31 seedlings (top panel, Fig. 5A). After 24 h of heat stress at 45 °C, Z31 leaves yellowed and plants showed severe damage and wilting, whereas transgenic plants retained greener leaves and exhibited less injury (middle panel, Fig. 5A). After 7 d of recovery, most Z31 control plants had died, while some transgenic plants recovered and continued to grow (bottom panel, Fig. 5A). The survival percentage of the three transgenic lines was significantly greater than that of Z31 (Fig. 5B), and their ion leakage was significantly reduced relative to the control (Fig. 5C).

### Overexpression of *AtSTS* reduces reactive oxygen species (ROS) accumulation under heat stress in Arabidopsis and maize

**Fig. 6:**
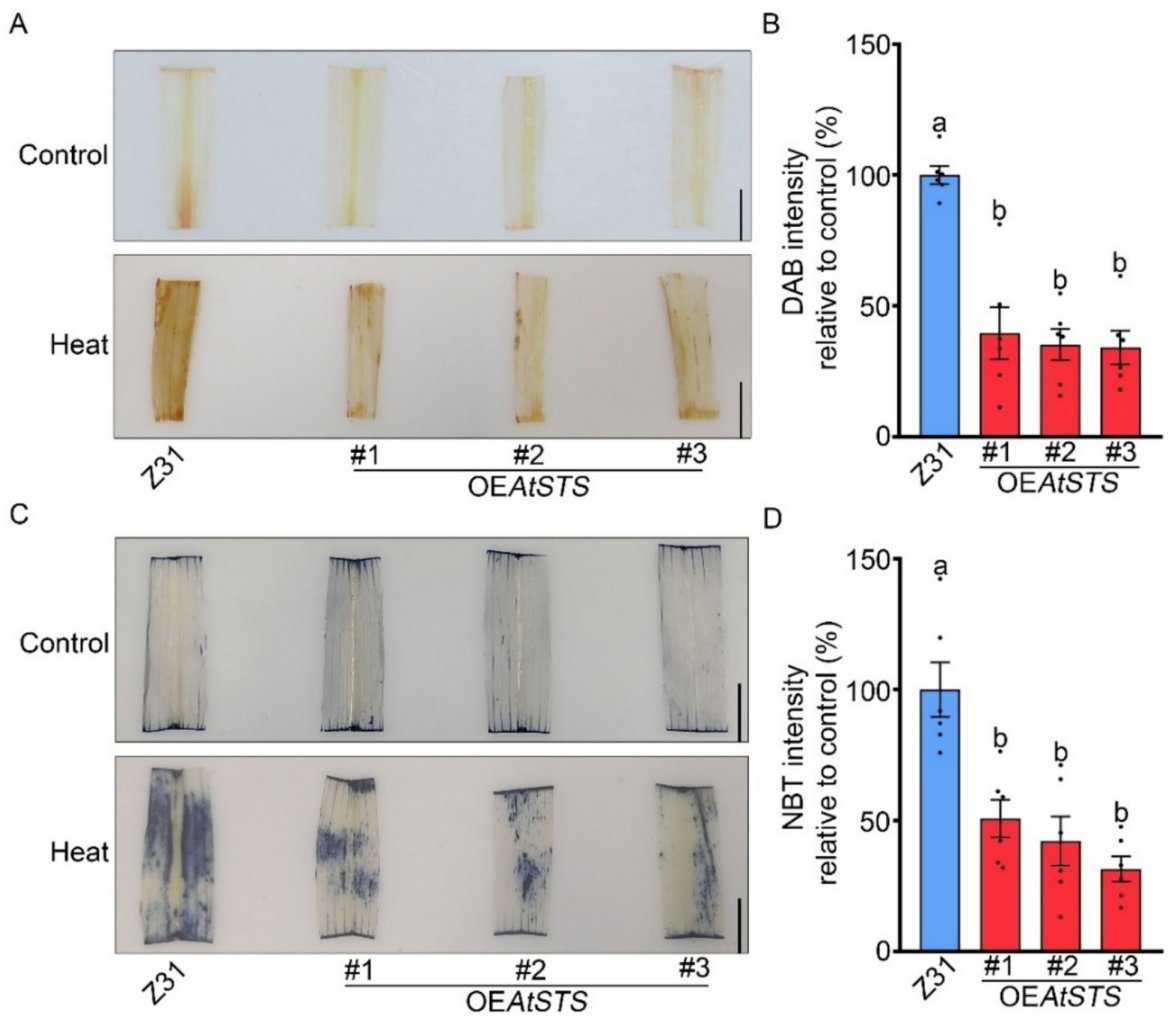
Overexpression of *AtSTS* reduces the accumulation of reactive oxygen species in maize seedling leaves under heat stress. **A** DAB (3,3′-diaminobenzidine tetrahydrochloride) staining of the leaves of Z31 and *AtSTS-*overexpressing (OE*AtSTS*) plants under control and heat-stress conditions. The scale bar = 1 cm. **B** Relative degree (%) of DAB staining observed under heat treatment. Each black dot represents one biological replicate; there are six biological replicates for each line. Values are means ± SEM. Different letters indicate significant differences (Duncan's test, *P* < 0.05). **C** NBT (nitroblue tetrazolium chloride) staining of the leaves of Z31 and OE*AtSTS* plants under control and heat-stress conditions. The scale bar = 1 cm. **D** Relative degree (%) of NBT staining observed under heat treatment. Each black dot represents one biological replicate; there are six biological replicates for each line. Values are means ± SEM. Different letters indicate significant differences (Duncan's test, *P* < 0.05)

To investigate whether the improved heat tolerance in *AtSTS*-overexpressing maize involves ROS mitigation, we assessed hydrogen peroxide (H₂O₂) and superoxide (O₂⁻) levels in Z31 and three *AtSTS*-transgenic lines before and after heat stress using 3,3′-diaminobenzidine tetrahydrochloride (DAB) and nitroblue tetrazolium chloride (NBT) histochemical staining, respectively. Under control conditions, ROS accumulation did not differ significantly among genotypes (upper panels, Fig. 6A and C). However, after heat stress, both staining methods revealed markedly stronger H₂O₂ and O₂⁻ signals in Z31 compared with the transgenic lines (lower panels, Fig. 6A and C). Quantification of staining intensity using ImageJ confirmed significantly greater ROS accumulation in stressed wild-type (WT) plants than in transgenic plants (Fig. 6B and D). Similarly, in Arabidopsis, no significant differences in ROS accumulation were observed among lines under normal conditions, whereas following heat stress, H₂O₂ and O₂⁻ signals in the leaves of WT Arabidopsis were markedly stronger than those in the three *AtSTS*-overexpressing Arabidopsis lines (Supplementary Fig. 5). These findings indicate that *AtSTS* overexpression enhances ROS scavenging capacity, thereby attenuating H₂O₂ and O₂⁻ accumulation under heat stress and consequently improving heat tolerance in both maize and Arabidopsis.

### Exogenous stachyose application enhances heat tolerance in both Arabidopsis and maize seedlings

To determine whether stachyose contributes critically to heat tolerance, 4-wk-old WT Arabidopsis seedlings were treated with a control solution (0.1% (v/v) Tween 20) or 5 mM stachyose solution containing 0.1% (v/v) Tween 20. Under normal conditions, both groups appeared morphologically similar (left panel, Supplementary Fig. 6 A). Following 3 d of heat stress at 40 °C, water-treated seedlings showed severe wilting and leaf yellowing, whereas stachyose-treated plants exhibited markedly less damage (middle panel, Supplementary Fig. 6 A). After a 7-d recovery period, most stachyose-treated seedlings recovered and resumed growth, in contrast to the high mortality in the control group (right panel, Supplementary Fig. 6 A). After heat stress, stachyose treatment significantly increased survival (Supplementary Fig. 6B) and reduced electrolyte leakage compared with the control group (Supplementary Fig. 6 C), indicating that exogenous stachyose enhances heat tolerance in Arabidopsis.

**Fig. 7:**
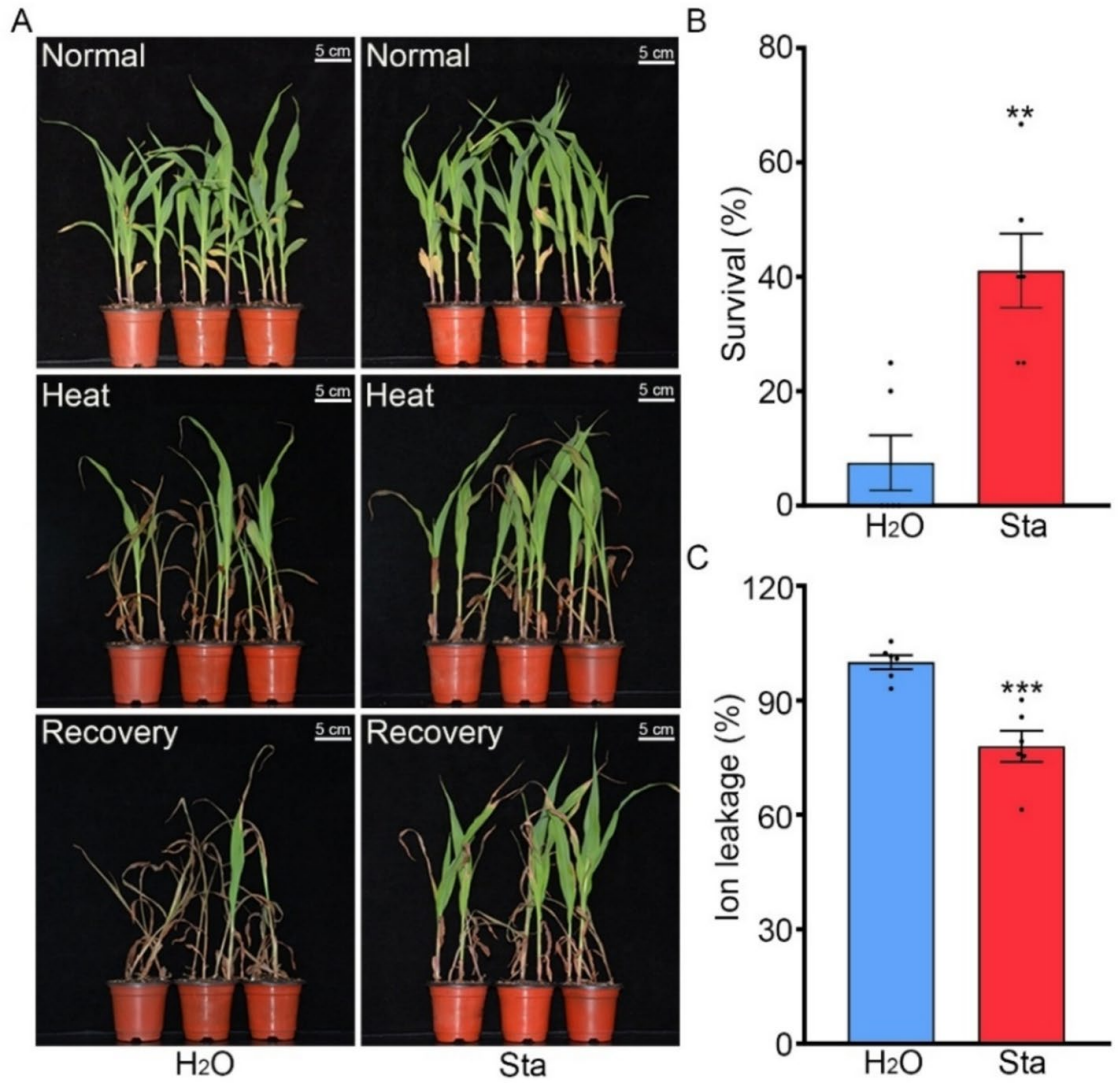
Exogenous application of stachyose enhances the heat tolerance of maize seedlings. **A** Morphological comparison of Z31 maize seedlings with exogenous application of Tween 20 (0.1% v/v) (H_2_O) and 10 mM stachyose with Tween 20 (0.1% v/v) (Sta) before heat stress (upper panel), after 3 d at 43℃ heat stress (middle panel), and 8 d of recovery (lower panel). The scale bar = 5 cm. **B** Comparison of survival in Z31 maize seedlings treated as in (A) with H_2_O or with exogenous Sta after 8 d of recovery following 3 d of 43℃ treatment. Each black dot represents one biological replicate (five plants); there are six biological replicates for each line. Values are means ± SEM. **P < 0.01 as compared by Student's *t*-test. **C** Comparison of the plasma membrane ion leakage in Z31 maize seedlings treated as in (A) with H_2_O or with exogenous Sta after 8 d of recovery following 3 d of 43℃ treatment. The second leaf from different plants was collected for testing of the electrolyte leakage. Each black dot represents one biological replicate (five plants); there are six biological replicates for each line. Values are means ± SEM. ***P < 0.001 as compared by Student's *t*-test

To further validate this effect in maize, V3-stage seedlings of the inbred line Z31 were treated with either a control solution 0.1% (v/v) Tween 20 or 10 mM stachyose solution containing 0.1% (v/v) Tween 20. Under normal conditions, both groups appeared morphologically similar (top panel, Fig. 7A). Following 4 d of heat stress at 43 °C, water-treated seedlings showed severe wilting and leaf yellowing, whereas stachyose-treated plants exhibited markedly less damage (middle panel, Fig. 7A). After an 8 d recovery period, most stachyose-treated seedlings recovered and resumed growth, in contrast to high mortality observed in the control group (bottom panel, Fig. 7A). The survival percentage of 10 mM stachyose-treated seedlings reached 41%, significantly higher than the 10% observed in controls (Fig. 7B). Consistent with this result, stachyose application significantly reduced heat-induced electrolyte leakage (Fig. 7C), confirming that exogenous stachyose enhances heat tolerance in maize. Importantly, exogenous stachyose treatment does not elevate endogenous galactinol or raffinose levels (data not shown), providing stronger evidence that stachyose alone is sufficient to confer heat protection, although the effects of exogenous application may not fully recapitulate the subcellular localization and temporal dynamics of endogenously synthesized stachyose.

### The field performance of *AtSTS*-overexpressing maize lines

The field performance of *AtSTS*-overexpressing maize lines was evaluated to determine whether constitutive expression of *AtSTS* affects plant growth, development, and yield. To this end, the parental line Z31 and three independent *AtSTS*-overexpressing lines were cultivated under field conditions and allowed to undergo natural pollination.

**Fig. 8:**
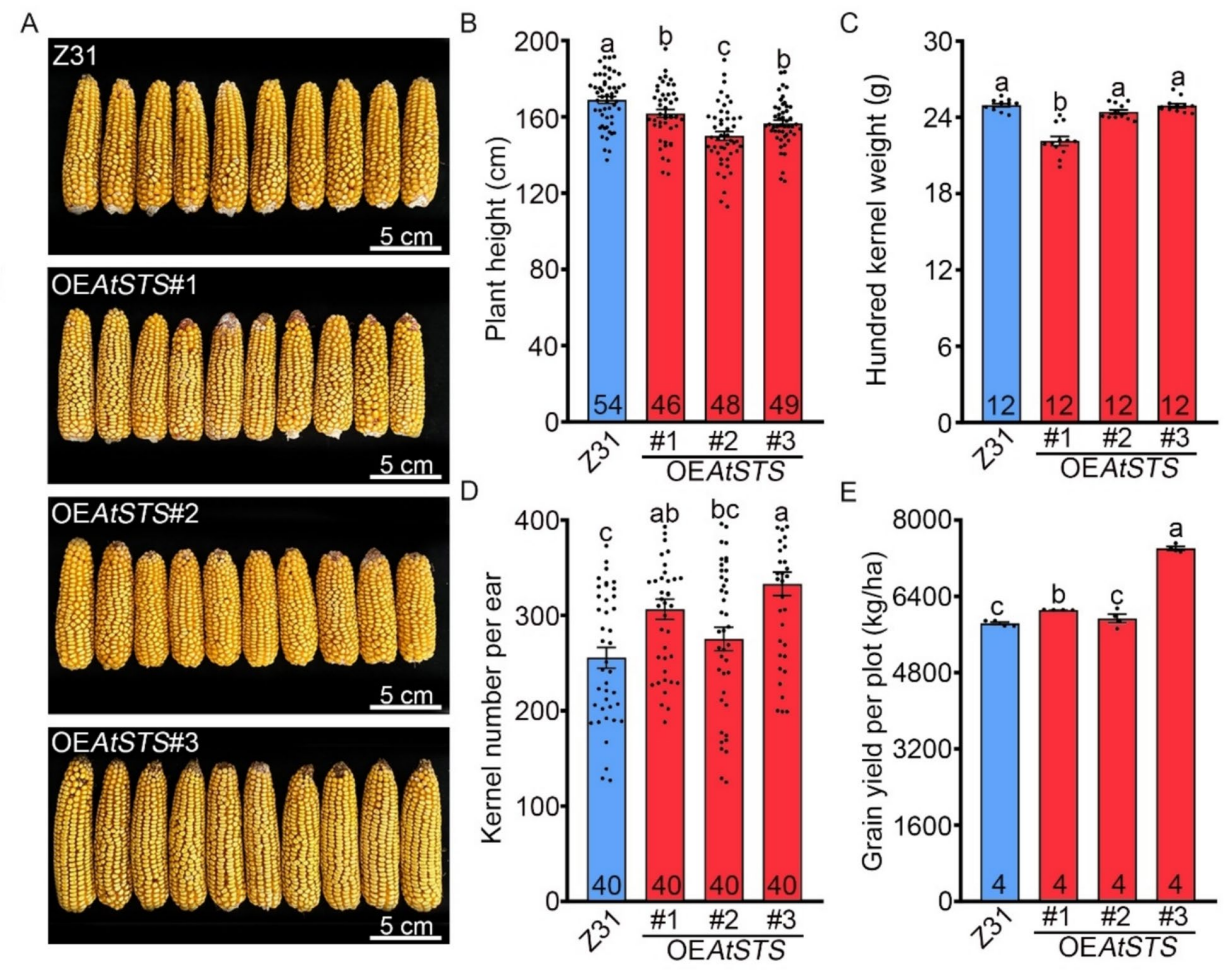
Overexpression of *AtSTS* does not have adverse effects on plant development and yield of maize. **A** Comparison of the ear phenotype between *AtSTS*-overexpressing (OE*AtSTS*) lines and the Z31 (Z31) control. The scale bar = 5 cm. **B**-**E** Comparison of plant height (**B**), hundred kernel weight (**C**), kernel number per ear (**D**) and the grain yield per plot (**E**) between OE*AtSTS* lines and the Z31 control line as given by the means ± SEM (n provided in the respective graph). Different letters indicate significant differences with Duncan's test using *P* < 0.05. The numbers in the bar graph bar indicate the replication numbers

No obvious differences in ear morphology or size were observed between Z31 and the transgenic lines (Fig. 8A), consistent with their similar genetic backgrounds following backcrossing and self-pollination (Supplementary Fig. 4). Although Z31 plants were slightly taller than the transgenic lines, the difference in mature plant height was approximately 4—10%, and no dwarfism or developmental abnormalities were observed in the *AtSTS*-overexpressing plants (Fig. 8B). Regarding yield components, no significant difference in 100-kernel weight was found between the OEAtSTS lines and control Z31 line, except that OEAtSTS line #1 showed a lower 100-kernel weight than the control (Fig. 8C). The kernel number per ear in OEAtSTS lines #1 and #3 was greater than that of the control, whereas no difference was observed between OEAtSTS line #2 and the control (Fig. 8D). The grain yield per plot of OEAtSTS lines #1 and #3 was significantly higher than that of the control, while no difference was detected between OEAtSTS line #2 and the control line (Fig. 8E). Notably, the yield increase in lines #1 and #3 under non-stress field conditions suggests either pleiotropic effects or natural variation, but the absence of a yield penalty in any line confirms that *AtSTS* overexpression does not adversely affect agronomic performance.These results suggest that AtSTS overexpression does not adversely affect maize growth, development, or yield under non-stress field conditions.

## Discussion

### Selective loss of *STACHYOSE SYNTHASE* before the evolution of *Zea* may compromise heat tolerance

In Arabidopsis, both the *STACHYOSE SYNTHASE* (*STS*) gene and stachyose accumulation are present, whereas neither STS activity nor stachyose accumulation has been detected in maize (Li et al. 2017). The loss of *STS* in the genus *Zea* appears to have occurred early in its evolutionary history rather than during domestication, as the maize precursor, *Zea luxurians*, had already lost the *STS* gene (Fig. 1). Besides the canonical RFO biosynthesis pathway, long-chain raffinose family oligosaccharides (RFOs) can also be synthesized by GALACTAN:GALACTAN GALACTOSYLTRANSFERASE (GGT). This enzyme catalyzes the transfer of an α-galactosyl residue from one RFO molecule to another, producing the next higher oligomer in the series, as demonstrated in *Ajuga reptans* (Haab and Keller 2002). To investigate whether a similar mechanism exists in maize, we performed a BLAST search of the maize genome using the *A. reptans* GGT (ArGGT) sequence as a query. No putative GGT ortholog was identified in maize. The closest related protein in maize is an ACIDIC ΑLPHA-GALACTOSIDASE (Supplementary Fig. 7). The absence of *STS* or GGT in maize, despite the retention of upstream enzymes such as RAFFINOSE SYNTHASE (RAFS), raises intriguing evolutionary questions. Several non-mutually exclusive explanations may account for this: raffinose alone may have been sufficient for protection from abiotic stress under the historical climatic conditions during *Zea* evolution; the metabolic cost of stachyose synthesis may have outweighed its benefits in certain environments (Okemo et al. 2021), or *Zea* species may have evolved alternative mechanisms for heat response. However, our findings demonstrate that restoring stachyose synthesis under current high-temperature stress significantly enhances heat tolerance by a general upregulation of RFO production, including the higher order oligosaccharide, stachyose (Fig. 5), suggesting that this evolutionary loss may represent a metabolic vulnerability in the face of rapidly warming climates.

### Extending the RFOs biosynthetic pathway in maize enhances its heat tolerance

RFOs are recognized as important molecules in plant heat stress responses (Gu et al. 2019). Maize has *GALACTINOL SYNTHASE* and *RAFFINOSE SYNTHASE* genes, the protein products from which have been demonstrated to positively influence abiotic stress tolerance (Nishizawa et al. 2008; Gu et al. 2016; Li et al. 2020; Liu et al. 2023). While the Arabidopsis* STACHYOSE SYNTHASE* gene product has been demonstrated to produce stachyose, it was unclear whether its introduction into maize, a species lacking an endogenous STS, would result in stachyose production. Our results demonstrate that *AtSTS* overexpression enables stachyose accumulation in maize, particularly under heat stress (Fig. 4D). However, a critical interpretative challenge arises from the observation that the transgenic lines also accumulate significantly higher levels of galactinol and raffinose under heat stress compared to wild-type controls (Fig. 4B, C), despite these metabolites being consumed as substrates for stachyose synthesis. This unexpected increase suggests that *AtSTS* overexpression may trigger broader metabolic feedback or flux redistribution within the RFO pathway, potentially involving upregulated substrate supply or reduced turnover of intermediates. Consequently, the improved heat tolerance observed in the *AtSTS*-overexpressing lines (Fig. 5) cannot be unambiguously attributed to stachyose alone. It may instead result from elevated levels of galactinol and raffinose—both established stress-protective metabolites (Nishizawa et al. 2008), or from synergistic effects among all three RFOs. Our exogenous stachyose application experiments (Fig. 7; Supplementary Fig. 6) partially address this confounding factor by demonstrating that stachyose alone, when applied exogenously, enhances heat tolerance in both maize and Arabidopsis. Nevertheless, this approach has inherent limitations. Exogenous application does not replicate the subcellular or temporal accumulation patterns of endogenously synthesized stachyose, nor does it account for potential metabolic interactions or feedback regulation that occur in transgenic plants.

We therefore acknowledge that the enhanced heat tolerance in *AtSTS*-overexpressing maize likely arises from a combination of elevated stachyose, raffinose, and galactinol, rather than from stachyose alone. Distinguishing among these possibilities would require additional genetic strategies, such as engineering lines that produce stachyose via a pathway that does not elevate upstream intermediates (e.g., using a galactinol-independent STACHYOSE SYNTHASE, if available); or conducting complementation experiments in Arabidopsis mutants. These experiments represent clear future directions. Until such experiments are performed, the precise contribution of stachyose to heat tolerance—relative to that of galactinol and raffinose—remains an open question.

Despite this limitation, the translational value of our approach is clear: *AtSTS* overexpression in maize leads to a coordinated elevation of multiple protective RFOs under heat stress, resulting in significantly improved thermotolerance without yield penalty under field conditions. Whether stachyose is the primary effector or part of a broader metabolic reconfiguration, the engineered extension of the RFO pathway represents a viable strategy for enhancing crop resilience to supraoptimal temperatures.

### Potential mechanisms of RFO-mediated heat tolerance of Arabidopsis and maize

The enhanced heat tolerance conferred by *AtSTS* overexpression can be attributed to multiple mechanisms, though the relative contributions of stachyose versus other RFOs remain to be disentangled. First, under heat stress, we observed significantly lower accumulation of hydrogen peroxide (H₂O₂) and superoxide (O₂⁻) in the leaves of *AtSTS*-overexpressing lines compared to control as detected by histochemical staining (DAB and NBT) (Fig. 6 and Supplementary Fig. 5). This aligns with the proposed role of oligosaccharides as ROS scavengers (Nishizawa et al. 2008). Notably, stachyose exhibits stronger hydroxyl radical scavenging capacity in vitro than raffinose or galactinol (Nishizawa-Yokoi et al. 2008), suggesting that stachyose may confer superior antioxidant protection. However, because our transgenic lines accumulate all three RFOs, we cannot rule out additive or synergistic effects among them.

Second, both *AtSTS* overexpression and exogenous stachyose application significantly reduced heat-induced electrolyte leakage from leaves (Figs. 5C and 7C; Supplementary Fig. 3 C and 6 C), indicating improved membrane thermostability. RFOs are known to interact with phospholipid bilayers via hydrogen bonds (Cacela and Hincha 2006). The fact that exogenous stachyose alone recapitulates this effect (Fig. 7C) provides stronger evidence for a direct membrane-protective role of stachyose, independent of galactinol or raffinose. Third, beyond its direct protective roles, stachyose could hypothetically function as a signaling molecule. Recent studies have shown that stachyose binds directly to HEAT SHOCK PROTEIN 90β (HSP90β) in the mouse intestine, triggering downstream signaling (Li et al. 2025). Given the high conservation of HSP90 structure and function among eukaryotes, it is tempting to speculate that stachyose accumulating in plant cells may interact with HSP90, potentially releasing HEAT SHOCK FACTOR A1s (HSFA1s) to initiate heat shock protein expression. However, we emphasize that this hypothesis is currently speculative and lacks direct experimental support in plants. Plant HSP90s have distinct regulatory mechanisms and client repertoires compared to their mammalian counterparts, and no evidence to date has demonstrated stachyose-HSP90 binding in plant systems. Therefore, we do not claim this as a demonstrated mechanism but rather as a testable hypothesis for future research. Targeted experiments—such as co-immunoprecipitation assays, or genetic interaction analyses between stachyose accumulation and plant HSP90 mutants, will be required to validate or refute this proposed signaling axis.

### Future application prospects for *AtSTS*-overexpressing maize

Against the backdrop of global warming and increasing extreme weather events, high-temperature-induced grain yield losses pose a significant threat to food security (Zhao et al. 2017). Our field trials demonstrated that, under non-stress conditions, *AtSTS*-overexpressing maize did not suffer a yield penalty relative to the control Z31 (Fig. 8E). Notably, two of the three transgenic lines showed significantly higher grain yield per plot compared to Z31 (Fig. 8E), suggesting that AtSTS overexpression may have positive pleiotropic effects or that the backcrossing process did not completely eliminate all background variation. Regardless, the absence of yield penalty in any line confirms the agronomic safety of this approach. Additionally, mature plant height decreased by approximately 4%–10%, which may enhance lodging resistance (Fig. 8B). This germplasm could potentially expand maize cultivation into regions traditionally considered unsuitable due to high temperatures during the growing season.

Nevertheless, a significant limitation of the current study is that all transgenic analyses were conducted exclusively in the maize inbred line Z31. While Z31 is a well-characterized laboratory line, it may not represent the genetic diversity found in elite commercial germplasm. The heat tolerance phenotype and stachyose accumulation profile observed here could be influenced by line-specific genetic backgrounds, metabolic networks, or epigenetic states. Therefore, the breeding relevance of this approach remains to be validated. Future studies should introduce the *AtSTS* transgene into multiple elite maize inbred lines representing different heterotic groups (e.g., Zheng58, Chang7-2, B73, Mo17) and assess their performance under heat stress in both controlled environments and multi-location field trials. Such validation would determine whether the observed heat tolerance is robust across diverse genetic backgrounds and whether any line-specific negative interactions occur.

Concurrently, this study confirmed that exogenous stachyose application improves maize heat tolerance (Fig. 7), offering a non-transgenic, complementary approach for crop stress protection. If stachyose proves to be stable, readily absorbable, and cost-effective, it holds promise for development as a natural biostimulant. Importantly, the exogenous application data—while not fully resolving the mechanistic confounding—demonstrate that stachyose alone is sufficient to enhance heat tolerance in wild-type plants, providing practical validation for its bioactivity independent of endogenous RFO elevation. Future research should focus on optimizing application techniques, evaluating the persistence of its protective effects, and exploring potential synergies with other stress-mitigating compounds. From a mechanistic standpoint, generating genetic tools to uncouple stachyose accumulation from upstream RFO elevation remains a priority for future studies.

## Conclusion

Expression of Arabidopsis *STACHYOSE SYNTHASE* in maize extends the raffinose family oligosaccharide pathway, enabling stachyose accumulation and enhancing heat tolerance by reducing oxidative damage, offering a promising strategy for crop improvement under rising global temperatures.

## Materials and methods

### Plant materials and growth conditions

The maize (*Zea mays L.*) inbred line, Z31, was maintained by the lab. Maize seeds were sown in sterilized soil composed of a 1:1 mixture of nutrient soil and vermiculite and cultured in a greenhouse under controlled conditions of 28℃, and a diurnal cycle of 16 h of light followed by 8 h of darkness.

The *AtSTS*-overexpressing Arabidopsis plants were previously generated and characterized (Li et al. 2017). Seeds of *A*. *thaliana* were surface sterilized and moist chilled at 4 °C for 2 d in the dark, then continued to germinate on GM agar plates [MS salt, 2.215 g L^−1^; 2-(N-morpholine)-ethansulfonic acid (MES), 2.05 mM; sucrose, 10 g L^−1^; pH = 5.7;] in a growth chamber under 16 h/8 h of light/dark at 22 °C. One wk later, the *A. thaliana* seedlings were transplanted into the sterilized soil, which is a mixture of nutrient soil and 2 g L^−1^ imidacloprid solution at a ratio of 1:1. The seedlings were subsequently cultivated in a greenhouse under the conditions of 22 °C, 16 h of light and 8 h of darkness.

### Maize transformation

To construct an *AtSTS*-overexpressing vector for maize transformation, the coding region of *AtSTS* was amplified by PCR from cDNA synthesized from RNA that was isolated from Arabidopsis seedling leaves using gene-specific primers OEAtSTS-F/R (Supplementary Table S1). The amplicon was purified and inserted into *Bam*H Ⅰ and *Hind* Ⅲ sites in the *pTF101.1* vector, and sequenced. The vector was transformed into immature embryos of maize inbred line Z31 using the *Agrobacterium*-mediated transformation method (Cho et al. 2014). Transgenic plants were screened by spraying with 1% (v/v) Basta herbicide. PCR was performed to characterize the *AtSTS* gene using primers OEAtSTS-F and OEAtSTS-R (Supplementary Table S1).

### Phylogenetic reconstruction of RFO-synthesizing enzyme genes

Amino acid sequences of ALKALINE ALPHA GALACTOSIDASE from *Chlamydomonas reinhardtii* (Cre16.g666334), *Physcomitrella patens* (Pp3c8_5920V3), *Selaginella moellendorffii* (XM_002961614); RAFFINOSE SYNTHASE from *Picea abies* (MA_5981g0010), *Amborella trichopoda* (XM_006840904), *Cucumis sativus* (Cucsa.098650, Cucsa.339920), *Prunus persica* (XM_007214907), *Glycine max* (Glyma.05G040300), *Arabidopsis thaliana* (AT5G40390), *Solanum lycopersicum* (Solyc03g112500), *Elaeis guineensis* (XM_010910277.2), *Ananas comosus* (Aco020323), *Setaria italica* (Seita.5G119800), *Sorghum bicolor* (Sobic.003G052300), *Brachypodium distachyon* (XM_003569223), *Oryza brachyantha* (XM_006643741), *Oryza sativa* (LOC_Os01g07530), *Zea mays* (GRMZM2G150906), *Zostera marina* (Zosma174g00010); and STACHYOSE SYNTHASE from *Picea abies* (MA_10436532g0010), *Amborella trichopoda* (XM_006841394.2), *Cucumis sativus* (Cucsa.204940), *Prunus persica* (XM 007221492), *Glycine max* (Glyma.19G217700), *Arabidopsis thaliana* (AT4G01970), *Solanum lycopersicum* (Solyc01g079300), *Elaeis guineensis* (XM_010933464), *Ananas comosus* (Aco013701), *Oryza brachyantha* (XM_006663594), *Setaria italica* (Seita.8G228200), *Sorghum bicolor* (Sobic.005G210100), *Brachypodium distachyon* (XM_003560828.2). All genes encode proteins demonstrated to possess the catalytic mechanism for which they were named or they were not included in the analysis.

### Sequence analysis of AtSTS

To identify the key structural domains of STS, the amino acid sequences of STS and RAFS from *A. thaliana*, *Zea mays*, and *Glycine max* were retrieved from the NCBI database (https://www.ncbi.nlm.nih.gov/). The sequences, AtSTS (NP_192106.3), SbSTS (XP_002451238.1), AtRAFS5 (NP_198855.1), and ZmRAFS (NP_001354805.1), were aligned using DNAMAN software (version 6.0.3).

### DNA extraction and PCR

Genomic DNA was isolated from maize leaves using the CTAB method (Porebski et al. 1997). The genotype of each transgenic plant was identified by PCR using *AtSTS* and *BAR* gene-specific primers (OEAtSTS-F/R; PTF-Bar-F/R; Supplementary Table 1).

### RNA extraction and quantitative RT-PCR

Total RNA was extracted from the leaves of three-leaf-stage seedlings of maize and four-week-old Arabidopsis following a protocol published previously (Li et al. 2017). Total RNA was extracted using RNAiso Plus (Takara, Japan). About 0.2 g of basal internodes from maize and Arabidopsis seedlings were ground into powder in liquid nitrogen and transferred to 800 μL of RNAiso Plus. The final extracted RNA was dissolved in 20 μL of diethylpyrocarbonate-treated water, and the RNA concentration was determined using a Nanodrop 200 spectrophotometer (Thermo Fisher Scientific, USA). Total RNA (2 μg) was used to synthesize cDNA using the Transcriptor First Strand cDNA Synthesis kit (Roche, Basel, Switzerland). Thirty-fold dilutions of cDNA were used as a template for real-time RT-PCR. The qRT-PCR was performed following a published protocol (Gu et al. 2013). *AtACTIN2* was employed as internal references for normalization of *AtSTS* expression in Arabidopsis. *ZmGAPDH* was employed as internal references for normalization of *AtSTS* expression in maize. The experiments were repeated three times with independent biological samples. For amplification of *ZmRAFS*, *AtSTS, ZmGAPDH* and *AtACTIN2* the primer sets, ZmRAFS-RT-F/R, SeqRT-AtSTS-F/R, ZmGAPDH-RT-F/R, and AtACTIN2-F/R (Supplementary Table 1), respectively, were used.

### Western blot

AtSTS polyclonal antibody was generated in the laboratory (Li et al. 2017). Western blot analysis of AtSTS protein accumulation in three-leaf-stage seedlings of maize and 4-week-old Arabidopsis leaves was performed following a published protocol (Gu et al. 2016). Protein detection used a Western Bright ECL Kit (Advansta, USA).

### Soluble carbohydrate extraction and HPLC-ELSD analysis of the sugar content

The second leaves of the V3 stage maize subjected to either normal conditions or 43 °C heat stress for 4 h was collected for sugar content analysis. Soluble sugar extraction followed a published protocol with minor revisions (Zhao et al. 2004). Maize leaves (0.5 g) were ground into powder in liquid nitrogen. One mL of 80% (v/v) ethanol containing 200 μg mL^−1^ lactose was added and homogenized to a slurry. Another 2 mL of 80% ethanol was used to wash the mortar followed by a further wash of 4 mL of 80% ethanol. These were combined and the suspensions were heated at 80℃ for 30 min, then centrifuged at 16,000 g to collect the supernatants. The tubes containing sugar extracts were opened and incubated in a water bath at 95℃ until the ethanol was evaporated. The remaining sugar solution (approximately 500 μL) was flash-frozen in liquid nitrogen and then lyophilized in a vacuum freeze-dryer for 4–6 h. The resulting powder was reconstituted in 300 μL of deionized water, filtered through a 0.22 μm filter to remove impurities, and the filtrate was stored at −80 °C. A Waters X-bridge amide column (Waters, USA) was washed by methanol/H_2_O (90:10) as the mobile phase at 0.5 mL·min^−1^ for separation of soluble sugar components when samples were introduced in a 10 μL volume. An evaporative light-scattering detector (ELSD; Waters 2424) was applied to monitor the sugar signal.

### Heat stress treatment of transgenic Arabidopsis

To test transgenic Arabidopsis heat stress tolerance, wild-type (WT) and *AtSTS*-overexpressing Arabidopsis seedlings from the same seedlot, grown at the same time, in the same place, were used. Seven days after the completion of germination, the seedlings were transplanted to soil (5 seedlings per pot) and cultured at 22 °C, 16-h/8-h light/dark diurnal cycles for 3 wk. Photographs were acquired to record the state of the seedlings prior to and immediately after heat stress at 43 °C for 36 h. The plants were photographed again following recovery at 22 °C for 6 d. The photographs were used to calculate survival. Ion leakage testing was then conducted.

### Heat stress treatment of transgenic maize

To test the heat stress tolerance of *AtSTS*-overexpressing maize lines, Z31 (control) and *AtSTS*-overexpressing maize seeds that had been harvested at the same time were used. After three d to complete germination in the dark, the seedlings were transplanted to soil (5 seedlings per pot) and grown to the V3 stage (Nleya et al. 2016) under 16 h light (900 ± 54 μmol m^−2^ s^−1^)/8 h dark at 28℃. Photographs were acquired to record the state of the plants prior to, and just after heat stress, and following one wk recovery. Seedlings of each genotype were transferred to a 16 h light (45℃, 900 ± 54 μmol m^−2^ s^−1^)/8 h dark (45℃) diurnal regime in an incubator and grown for 1 d for heat stress treatment. The 45 °C temperature was chosen based on preliminary experiments showing that 24 h at 45 °C caused substantial but not complete damage to wild-type Z31 seedlings, allowing discrimination of enhanced tolerance in transgenic lines. The plants were then allowed to recover at 16 h light (900 ± 54 μmol m^−2^ s^−1^)/8 h dark at constant 28℃ for 7 d. Photos were taken to record the state of the plants after recovery, then survival was recorded. Ion leakage testing was subsequently conducted.

### Electrolyte leakage measurement

After recovery, the second leaf of maize or all the leaves of Arabidopsis were harvested and the fresh weight taken before they were placed into a test tube containing 15 mL of deionized water. After vacuum filtration for 30 min, the tube was shaken at 80 rpm for 1 h at 25 °C. Then the conductivities (K1) of the solutions were determined. The leaves were subsequently boiled in the same deionized water for 30 min. After cooling to room temperature, the conductivities (K2) were determined again. The experiments were repeated three times with independent biological samples. The ratios of K1/K2 were calculated and used to evaluate the degree of electrolyte leakage. Raw K1/K2 ratios are reported; for comparison across experiments, values were normalized to the wild-type control within each experiment (set to 100%).

### Exogenous stachyose treatment

To assess the effect of exogenous stachyose on heat tolerance in maize seedlings, seedlings of the inbred line Z31 at the V3 stage were divided into a control group and an experimental group, with six pots per group and five seedlings per pot. The control group received foliar sprays of 80 mL of an aqueous solution containing 0.1% (v/v) Tween-20, while the experimental groups was treated with an equal volume of 10 mM stachyose solution containing 0.1% (v/v) Tween-20, until all leaves were completely wetted. All seedlings were subjected to heat stress in a growth chamber for 4 d under cycles of 16 h light (43 °C, 900 ± 54 μmol m⁻^2^ s⁻^1^) and 8 h dark (43 °C), During the stress period, seedlings received foliar sprays of 80 mL every 24 h, for a total of 4 times. Following the stress treatment, the seedlings were allowed to recover for 7 days under 16 h light (900 ± 54 μmol m^−2^ s^−1^)/8 h dark conditions at 28℃. After recovery, plant survival was recorded, and ion leakage was measured.

To assess the effect of exogenous stachyose on heat stress in Arabidopsis seedlings, 4-wk-old wild type (WT) seedlings were divided into a control group and an experimental group, with six pots per group and five seedlings per pot. The control group received foliar sprays of 25 mL of an aqueous solution containing 0.1% (v/v) Tween-20, while the experimental group was treated with an equal volume of 5 mM stachyose solution also containing 0.1% (v/v) Tween-20. All seedlings were subjected to heat stress in a growth chamber under for 3 d under cycles of 16 h light and 8 h at 40 °C. During the stress period, seedlings received daily foliar sprays of 25 mL every 24 h, for a total of 3 times. Following the stress treatment, the seedlings were allowed to recovery for 7 days under 16 h light/8 h dark conditions at 22℃. After recovery, plant survival was recorded, and the ion leakage was measured.

### Histochemical staining and determination of physiological indexes

To detect H₂O₂ and O₂⁻ accumulation, leaves were collected from 4-wk-old WT and *AtSTS*-overexpressing Arabidopsis plants (grown at 16 h light/8 h dark at 22℃), as well as from V3-stage Z31 and *AtSTS*-overexpressing maize plants (grown at 16 h light (900 ± 54 μmol m^−2^ s^−1^)/8 h dark at 25℃). For each genotype, two groups were established: a normal growth control (see conditions detailed above) and a heat stress treatment group. Seedlings in the heat stress group were subjected to 43 °C (Arabidopsis) or 45 °C (maize) for 12 h, while control seedlings remained under normal growth conditions. After heat treatment, leaves of appropriate size (maintain consistency among all the strains) were excised and vacuum-infiltrated for 20 min in either a 0.5 mg mL^−1^ (pH 3.8) solution of 3,3'-diaminobenzidine tetrahydrochloride (DAB) or a 0.3 mg mL^−1^ solution of nitroblue tetrazolium chloride (NBT). The samples were then incubated in the staining solutions at room temperature in darkness for 12 h. To remove chlorophyll interference, the stained samples were transferred to 80% ethanol and decolorized in an 80 °C water bath with several changes of solution until the tissues were completely bleached. The decolorized samples were placed on a white background for observation and photography. Quantitative analysis of the staining intensity was performed using Image J software (http://www.imagej.nih.gov/ij/). Six biological replicates were analyzed for each genotype and treatment combination.

### Determination of maize agronomic traits

Z31 and *AtSTS*-overexpressing maize plants were cultivated at an experimental farm in Sanya at a planting density of 82, 500 plants per hectare. Plant height was measured after flowering. After harvest, grain weight was recorded, and grain moisture content was determined accordingly. Final yield was calculated and adjusted to a standard moisture content of 14%.

### Statistical analysis

The data were analyzed by one-way ANOVA (Duncan’s test) or Student’s *t*-test using SPSS 23.

## Supplementary Information

Supplementary Material 1: Supplementary Figure 1. Phylogenetic analysis of raffinose family oligosaccharide (RFO) SYNTHASE-related proteins across representative plant species. Supplementary Figure 2. Arabidopsis* AtSTS *responds to heat stress. Supplementary Figure 3. Overexpression of *AtSTS* enhances the heat tolerance of Arabidopsis seedlings. Supplementary Figure 4. Schematic of the transgenic maize breeding scheme. Supplementary Figure 5. Overexpression of *AtSTS* reduces the accumulation of reactive oxygen species in Arabidopsis seedling leaves under heat stress. Supplementary Figure 6. Exogenous application of stachyose enhances the heat tolerance of Arabidopsis seedlings. Supplementary Figure 7. Absence of GGT in maize. Supplementary Table 1. Primers used in this study.

## Data Availability

All relevant data can be found within the manuscript and its supporting information.

## Abbreviations

AGA: ALKALINE ALPHA-GALACTOSIDASE
DAB: Diaminobenzidine
GAPDH: GLYCERALDEHYDE-3-PHOSPHATE DEHYDROGENASE
GGT: GALACTAN GALACTOSYLTRANSFERASE
GOLS: GALACTINOL SYNTHASE
RAFS: RAFFINOSE SYNTHASE
RFOs: Raffinose family oligosaccharides
ROS: Reactive oxygen species
STS: STACHYOSE SYNTHASE
NBT: Nitroblue tetrazolium chloride
VES: VERBASCOSE SYNTHASE
