# Supplementary material for: Reconstruction of the raffinose family oligosaccharide pathway in maize improves heat stress tolerance
Source: Stress Biol. 2026 Jul 16;6(1):49. doi: 10.1007/s44154-026-00326-0 (PMC13376108; doi:10.1007/s44154-026-00326-0)
Supplement: Supplementary file 1 — Supplementary Material 1: Supplementary Figure 1. Phylogenetic analysis of raffinose family oligosaccharide (RFO) SYNTHASE-related proteins across representative plant species. Supplementary Figure 2. Arabidopsis AtSTS responds to heat stress. Supplementary Figure 3. Overexpression of AtSTS enhances the heat tolerance of Arabidopsis seedlings. Supplementary Figure 4. Schematic of the transgenic maize breeding scheme. Supplementary Figure 5. Overexpression of AtSTS reduces the accumulation of reactive oxygen species in Arabidopsis seedling leaves under heat stress. Supplementary Figure 6. Exogenous application of stachyose enhances the heat tolerance of Arabidopsis seedlings. Supplementary Figure 7. Absence of GGT in maize. Supplementary Table 1. Primers used in this study. [file 44154_2026_326_MOESM1_ESM.docx]

**Supplementary information**

TITLE: Reconstruction of the Raffinose Family Oligosaccharide Pathway in Maize Improves Heat Stress Tolerance

Zhongchun Dong^1^• Tao Li^1,2^• Xudong Li^1,3^•Dan Li^1^•Xianfei Shi^1^• Jiahao Chai^1^• Lynnette M.A. Dirk^4^ •A. Bruce Downie^4^ •Tianyong Zhao^1*^

^1^ State Key Laboratory of Crop Stress Resistance and High-Efficiency Production, College of Life Sciences, Northwest A&F University, Yangling, Shaanxi 712100, China

^2^State Key Laboratory of Wheat and Maize Crop Science, Collaborative Innovation Center of Henan Grain Crops, College of Life Science, Henan Agricultural University, Zhengzhou, Henan, 450002, China.

^3^College of agriculture and bioengineering (Peony college), Heze University. 2269 Daxue Road, Heze 274015, Shandong, P.R. China.

^4^ Department of Horticulture, Seed Biology, Martin-Gatton College of Agriculture, Food and Environment, University of Kentucky, Lexington, KY 40546, USA

*Corresponding author

Tianyong Zhao, Ph.D

Department of Biochemistry and Molecular Biology

College of Life Sciences, Northwest A&F University

Yangling, Shaanxi 712100, China

Email: tzhao2@nwafu.edu.cn

Tel: 86-13324517154


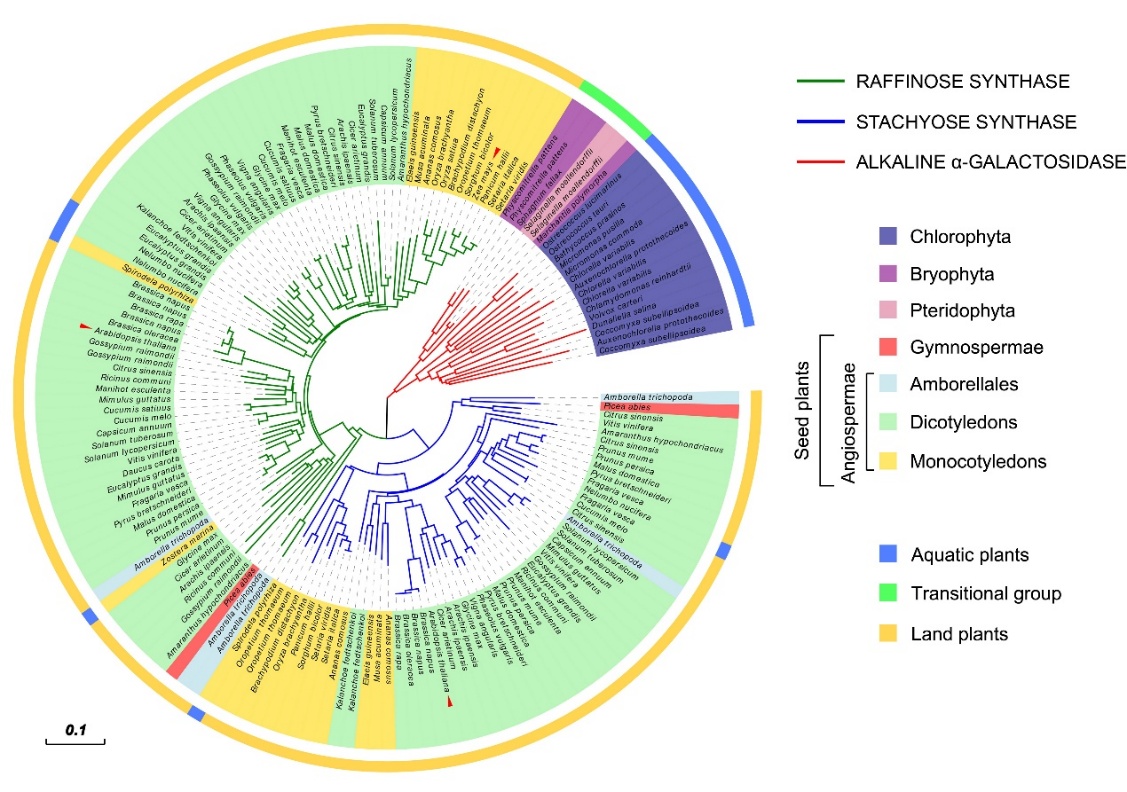


**Supplementary Figure 1. Phylogenetic analysis of raffinose family oligosaccharide (RFO) SYNTHASE-related proteins across representative plant species.**

ZmRAFS, AtRAFS (AtRS5), and AtSTS (AtRS4) were used as query sequences to identify homologous proteins by BLASTP searches (E-value cutoff = 1e−10) against publicly available databases, including NCBI, JGI Phytozome, and CottonGen. A total of 62 species were sampled, including 12 green algae (*Chlamydomonas reinhardtii, Coccomyxa subellipsoidea, Dunaliella salina, Monoraphidium neglectum, Volvox carteri, Bathycoccus prasinos, Micromonas commoda, Micromonas pusilla, Ostreococcus lucimarinus, Ostreococcus tauri, Auxenochlorella protothecoides,* and *Chlorella variabilis*), 3 bryophytes (*Marchantia polymorpha, Physcomitrella patens,* and *Sphagnum fallax*), 1 lycophyte lineage (*Selaginella moellendorffii*), 1 gymnosperm (*Picea abies*), 31 dicotyledonous species, and 14 monocotyledonous species. Multiple sequence alignment was performed using ClustalW, and the phylogenetic tree was constructed using the Neighbor-Joining method with 1,000 bootstrap replicates. Branches are color-coded according to protein function: RAFFINOSE SYNTHASE (RAFS, green), STACHYOSE SYNTHASE (STS, blue), and alkaline α-galactosidase (AGA, red). Colored strips surrounding the tree indicate the taxonomic distribution of species, including chlorophyta, bryophytes, pteridophytes, gymnosperms, and angiosperms. Within angiosperms, Amborellales represents basal angiosperms, while monocotyledons and dicotyledons represent major flowering plant lineages. These groupings reflect evolutionary clades rather than strictly ranked taxonomic levels. The outermost ring indicates ecological adaptation categories, including aquatic plants, transitional lineages, and terrestrial plants. Gray dashed lines indicate guide lines for aligning tip labels. The scale bar represents amino acid substitutions per site. Red arrows indicate representative proteins, including ZmRAFS from maize (inbred line B73) and AtRAFS and AtSTS from *Arabidopsis*.


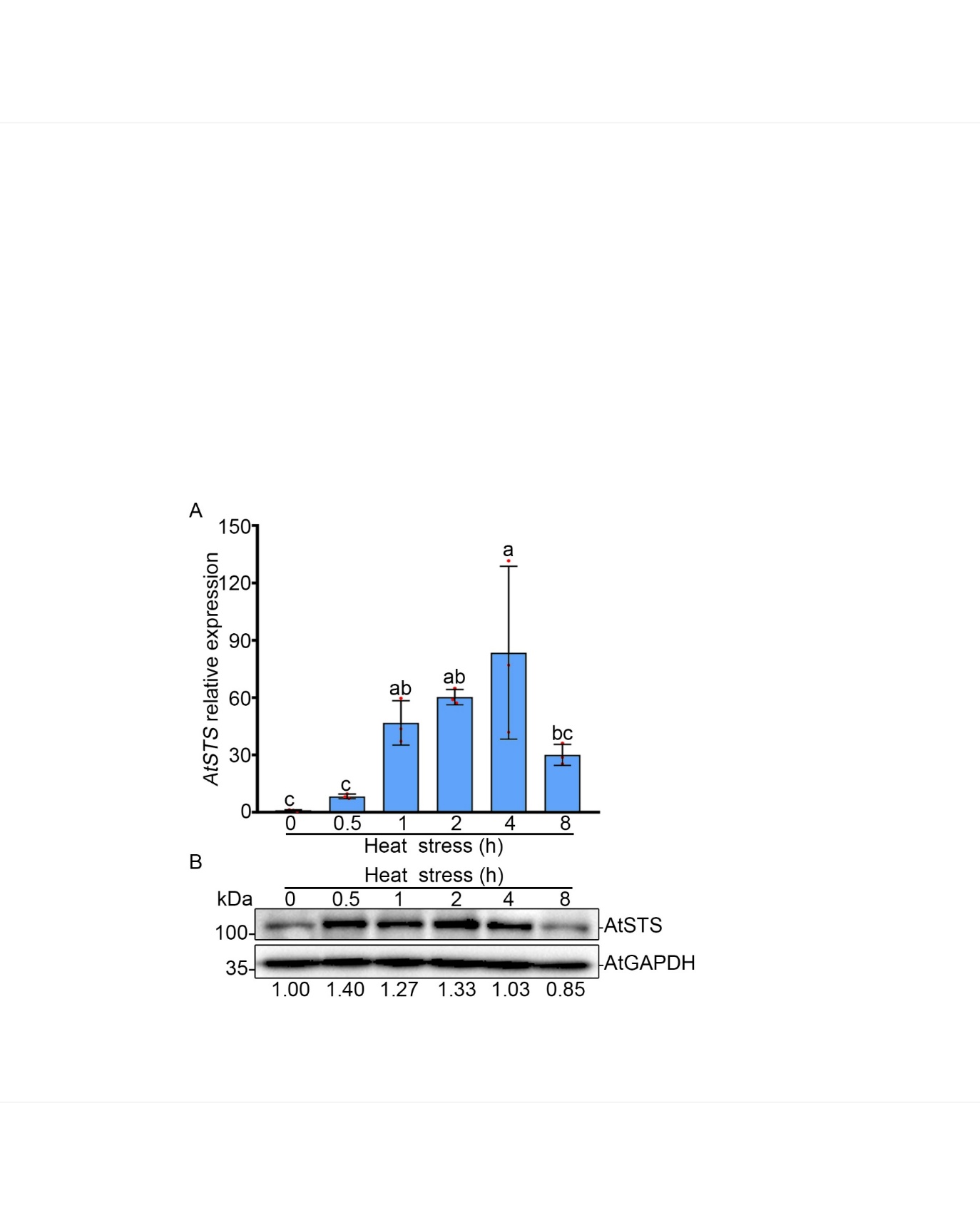


**Supplementary Figure 2. *Arabidopsis* *AtSTS* is induced by heat stress.**

(A) Quantitative RT-PCR characterization of the *AtSTS* mRNA accumulation in the leaves of *Arabidopsis* seedlings after heat stress at 43°C for 0, 0.5, 1, 2, 4, and 8 h. *AtACTIN2* accumulation was used as the internal control. Each red dot represents one biological replicate; there are three biological replicates for each line. Values are means ± SEM. Different letters indicate significant difference (Duncan test, *P* < 0.05).

(B) Western blot analysis of the AtSTS protein accumulation in the leaves of *Arabidopsis* seedlings after heat stress at 43°C for 0, 0.5, 1, 2, 4, and 8 h. AtGAPDH was used as the internal reference. Band volumes (arbitrary units) were assessed using Image J. The AtSTS amounts were normalized by AtGAPDH amounts, represented by the band volume ratios under the blots.


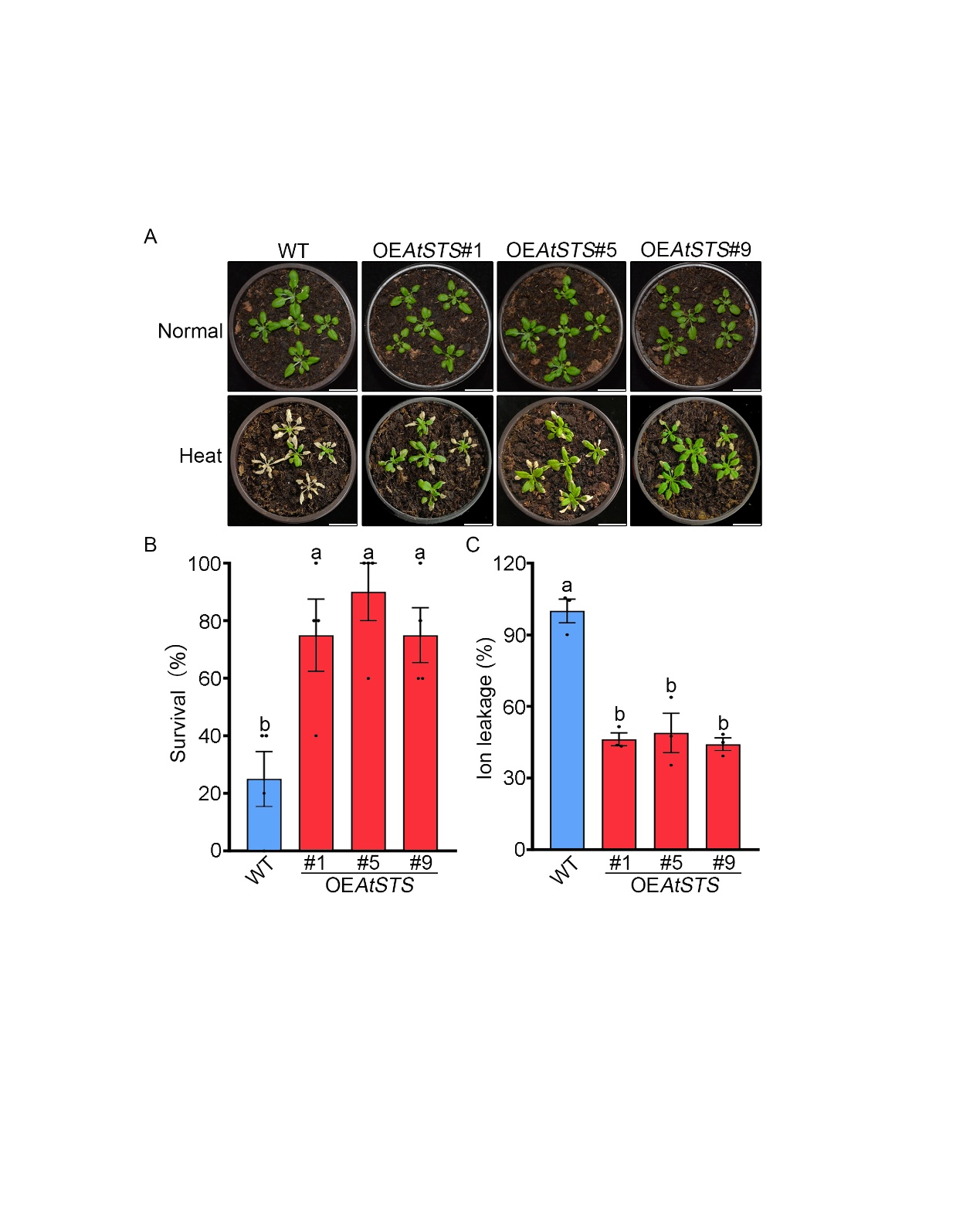


**Supplementary Figure 3. Overexpression of *AtSTS* enhances the heat tolerance of *Arabidopsis* seedlings.**

(A) Morphology comparisons between control *Arabidopsis* (WT) seedlings and *AtSTS*-overexpressing (OE*AtSTS*) *Arabidopsis* seedlings before heat stress (upper panel), after 36 h of 43℃ heat stress and 6 d after recovering (lower panel). The scale bar = 1 cm.

(B) Comparison of the survival percentage between WT and OE*AtSTS* *Arabidopsis* seedlings after heat stress. Each black dot represents one biological replicate (of five plants each); there are four biological replicates for each line. Data are means ± SEM. Different letters indicate significant differences (Duncan's test, *P* < 0.05).

(C) Comparison of the plasma membrane ion leakage between WT and OE*AtSTS* *Arabidopsis* seedlings after heat stress and 6 d recovery. All leaves from different surviving plants were collected for testing of the electrolyte leakage. Each black dot represents one biological replicate (of six plants each); there are three biological replicates for each line. Data are means ± SEM. Different letters indicate significant differences (Duncan's test, *P* < 0.05).


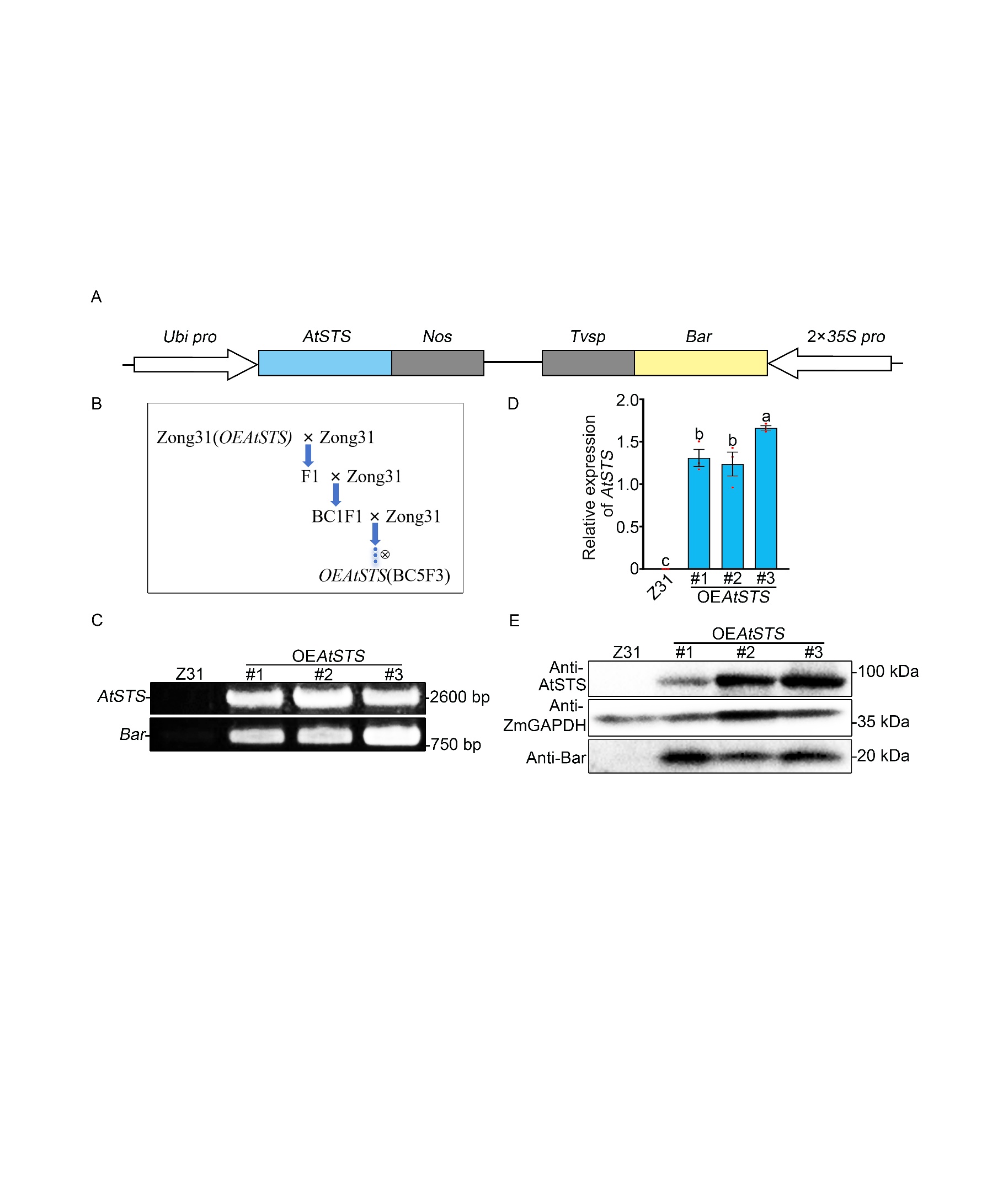


**Supplementary Figure 4. Schematic of the transgenic maize breeding scheme.**

Using the maize inbred line Zong31 as the paternal parent, three genetically stable transgenic homozygous lines were ultimately obtained through multiple generations of backcrossing and selfing. BC5F3 indicates 5 generations of backcrossing and 3 generations of selfing.


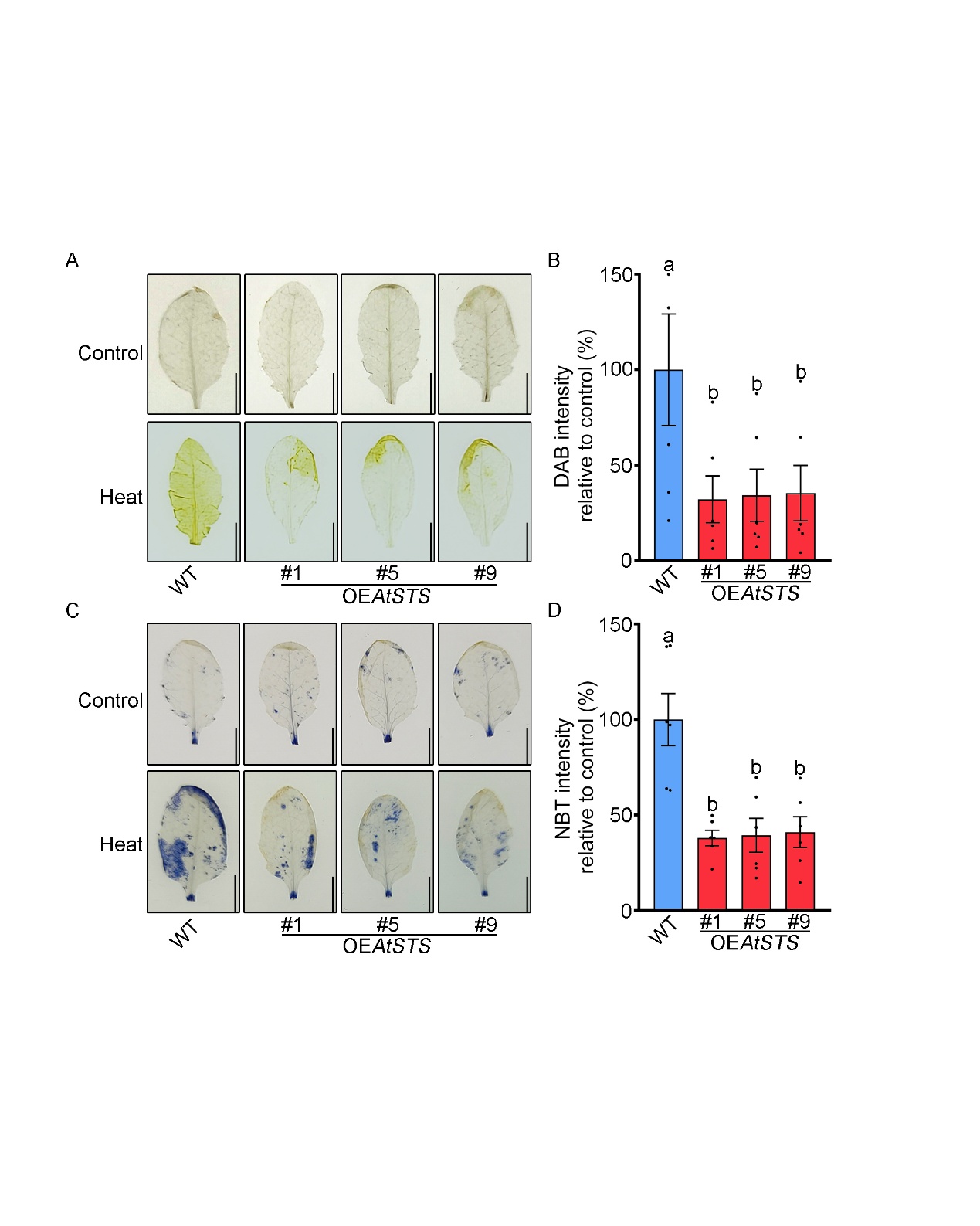


**Supplementary Figure 5. Overexpression of *AtSTS* reduces the accumulation of reactive oxygen species in Arabidopsis seedling leaves under heat stress.**

(A) Staining with 3,3′-diaminobenzidine tetrahydrochloride (DAB) of leaves from 4-wk-old wild-type (WT) and *AtSTS*-overexpressing (OE*AtSTS*) *Arabidopsis* plants before and after heat stress. The scale bar = 5 mm.

(B) Relative degree (%) of DAB staining under heat treatment. Data are means ± SEM (n = 6). Different letters indicate significant differences (Duncan's test, *P* < 0.05).

(C) Staining with nitroblue tetrazolium chloride (NBT) of leaves from 4-wk-old WT and OE*AtSTS Arabidopsis* plants before and after heat stress. The scale bar = 5 mm.

(D) Relative degree (%) of NBT staining under heat treatment. Data are means ± SEM (n = 6). Different letters indicate significant differences (Duncan's test, *P* < 0.05).

**
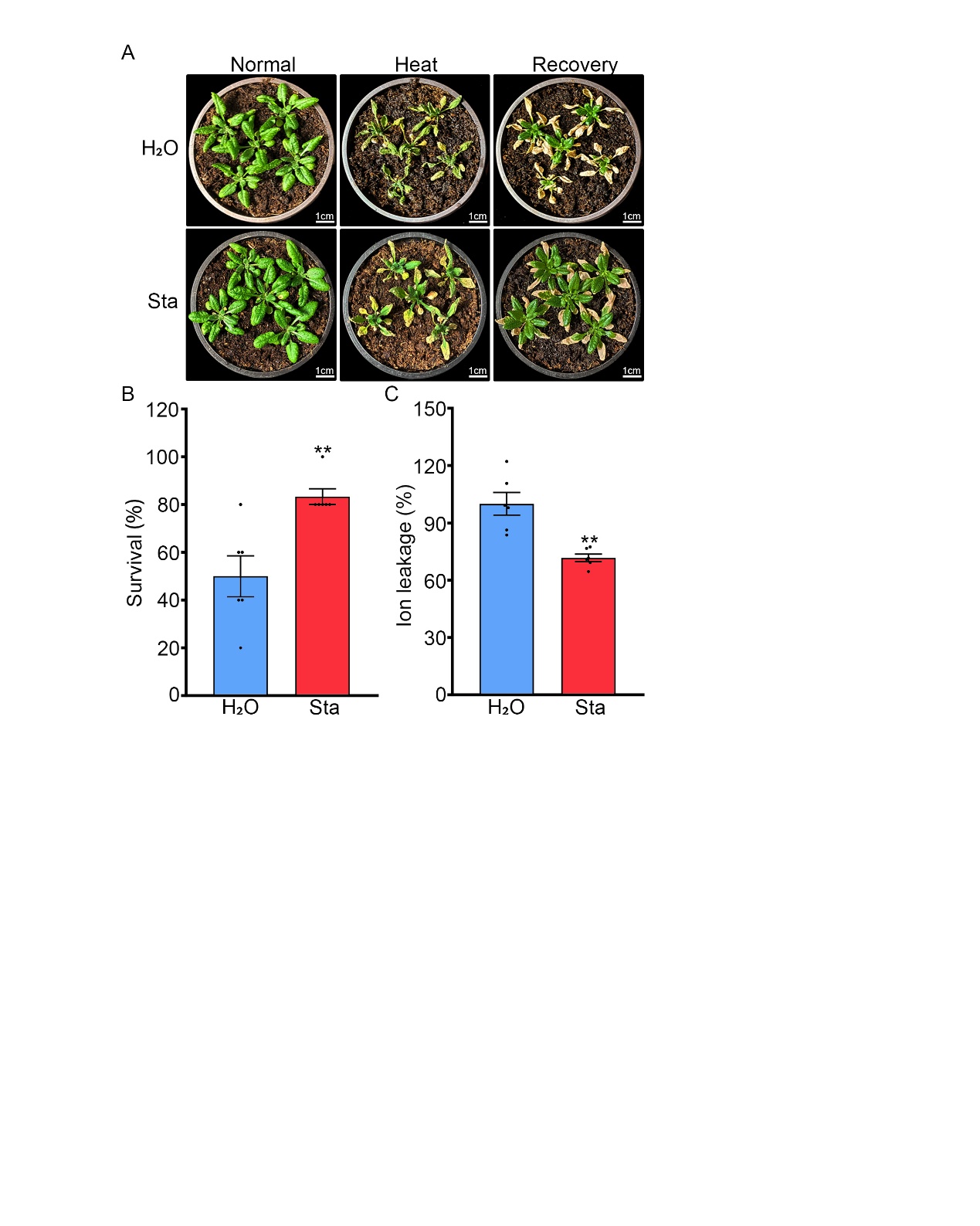
**

**Supplementary Figure 6. Exogenous application of stachyose enhances the heat tolerance of *Arabidopsis* seedlings.**

(A) Morphological comparison of WT *Arabidopsis* seedlings with exogenous application of Tween 20 (0.1% v/v) (H_2_O) and 5 mM stachyose with Tween 20 (0.1% v/v) (Sta) before heat stress (left panel), after 3 d of 40℃ heat stress (middle panel), and 7 d of recovery at 22°C (right panel). The scale bar = 1 cm.

(B) Comparison of survival of WT *Arabidopsis* seedlings treated as in (A) with H_2_O or with exogenous Sta after 7 d of recovery. Each black dot represents one biological replicate (5 plants); there are six biological replicates for each line. Values are means ± SEM. ***P* < 0.01 as compared by Student's *t*-test.

(C) Comparison of the electric conductivity of WT *Arabidopsis* seedlings treated as in (A) with H_2_O or with exogenous Sta after 7 d of recovery. All leaves from different plants were collected for testing of the electric conductivity. Each black dot represents one biological replicate (5 plants); there are six biological replicates for each line. Values are means ± SEM. ***P* < 0.01 as compared by Student's *t*-test.

**
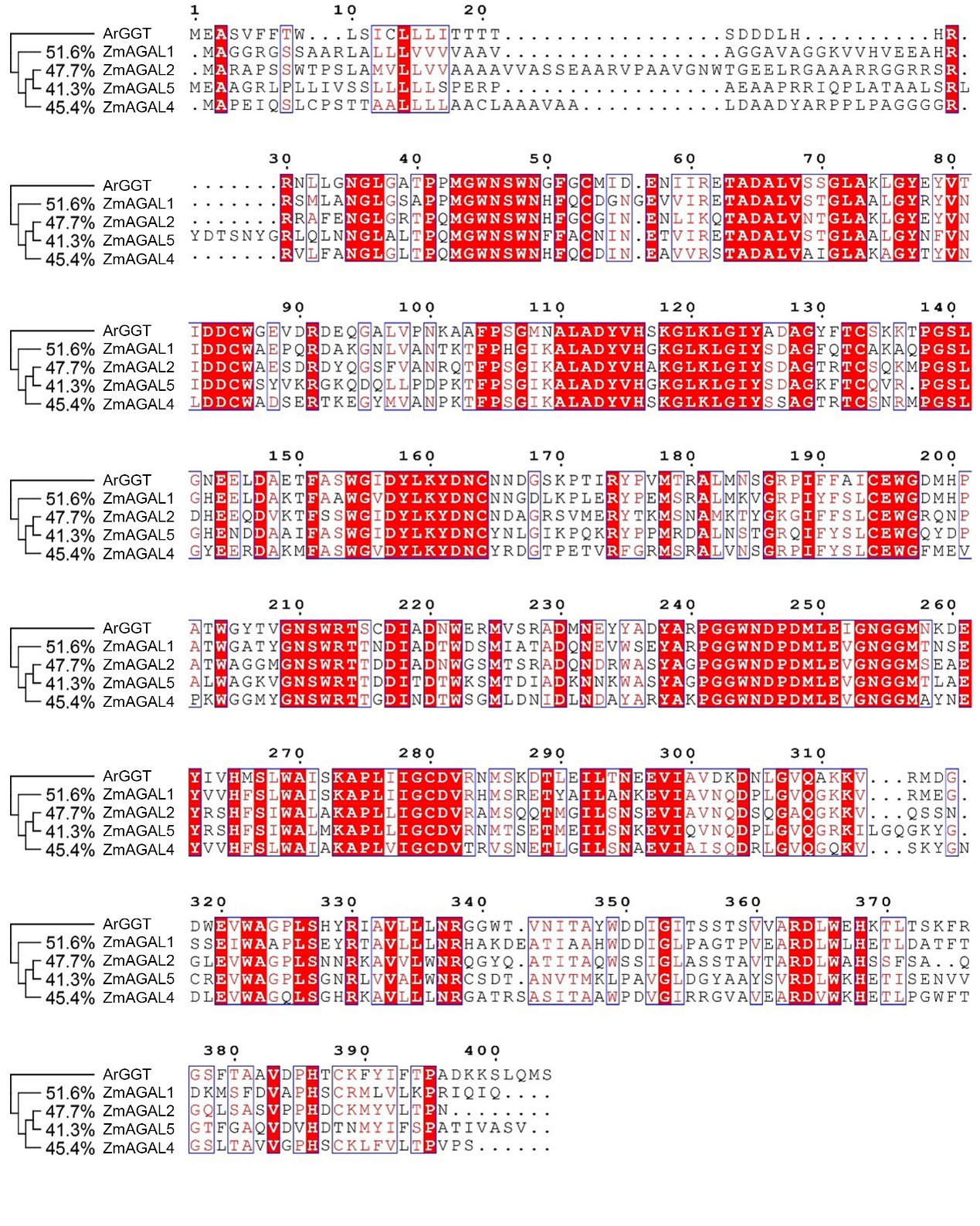
**

**Supplementary Figure 7. Absence of GGT in maize.**

Using the GGT protein sequence from *Ajuga reptans* (ArGGT, AAR02007.1) as a query, a search was performed against the maize B73 reference genome; however, no protein sequence with high homology to ArGGT was identified. The 4 proteins: ACIDIC ALPHA-GALACTOSIDASEs (ZmAGAL1: GRMZM2G095126, ZmAGAL2: GRMZM2G346455, ZmAGA5: GRMZM2G053554, ZmAGAL4: GRMZM2G146463) in maize showed the highest similarity to GGT. The left panel shows the phylogenetic relationship between ArGGT and four maize AGAL enzymes. Sequence alignment was performed using the MUSCLE method, and the phylogenetic tree was constructed using the maximum likelihood method (bootstrap = 1,000). The percentage in the figure indicates the sequence similarity among the four maize AGAL enzymes and the ArGGT.

**Supplementary Table 1. Primers used in this study.**

| **Primer name** | **Sequence (5'-3')** |
| --- | --- |
| OEAtSTS-F | ATGGCTCCACTTCACGAAT |
| OEAtSTS-R | TTAAAAGGTGAAAGACAGATG |
| PTF-Bar-F | ATGGAACAAGGGCAGAAGATT |
| PTF-Bar-R | GCACCATCGTCAACCACTAC |
| SeqRT-AtSTS-F | GACAAGGTTGTAGAAGCTGGGATT |
| SeqRT-AtSTS-R | TGTGGCAAGGAAGAAGAACTCAT |
| AtACTIN2-RTF | CTTAACCCAAAGGCCAACAGA |
| AtACTIN2-RTR | GCAAGGTCAAGACGGAGGAT |
| ZmRAFS-RTF | CGTGGGACGCCTTCTACCT |
| ZmRAFS-RTR | CCCTGCTTGTACTCCCTGAAC |
| ZmGAPDH-RTF | CCCTTCATCACCACGGACTAC |
| ZmGAPDH-RTR | AACCTTCTTGGCACCACCCT |
